# Re-Evaluation of Reportedly Metal Tolerant *Arabidopsis thaliana* Accessions

**DOI:** 10.1371/journal.pone.0130679

**Published:** 2016-07-28

**Authors:** Macarena Silva-Guzman, Charles Addo-Quaye, Brian P. Dilkes

**Affiliations:** Department of Horticulture and Landscape Architecture, Purdue University, West Lafayette, Indiana, United States of America; Iwate University, JAPAN

## Abstract

Santa Clara, Limeport, and Berkeley are *Arabidopsis thaliana* accessions previously identified as diversely metal resistant. Yet these same accessions were determined to be genetically indistinguishable from the metal sensitive Col-0. We robustly tested tolerance for Zn, Ni and Cu, and genetic relatedness by growing these accessions under a range of Ni, Zn and Cu concentrations for three durations in multiple replicates. Neither metal resistance nor variance in growth were detected between them and Col-0. We re-sequenced the genomes of these accessions and all stocks available for each accession. In all cases they were nearly indistinguishable from the standard laboratory accession Col-0. As Santa Clara was allegedly collected from the Jasper Ridge serpentine outcrop in California, USA we investigated the possibility of extant *A*. *thaliana* populations adapted to serpentine soils. Botanically vouchered Arabidopsis accessions in the Jepson database were overlaid with soil maps of California. This provided no evidence of *A*. *thaliana* collections from serpentine sites in California. Thus, our work demonstrates that the Santa Clara, Berkeley and Limeport accessions are not metal tolerant, not genetically distinct from Col-0, and that there are no known serpentine adapted populations or accessions of *A*. *thaliana*.

## Introduction

*Arabidopsis thaliana* is widely used as a model organism in plant sciences for its high number of seeds per plant, short life cycle, small genome, and self-fertility. The first *A*. *thaliana* accession was described in the sixteenth century [1] and careful curation of collections has resulted in 5683 currently available accessions at the Arabidopsis Biological Resource Center (ABRC; abrc.osu.edu). These were collected from locations that differ in temperature, day length, precipitation levels, salinity, altitude, and biotic pressures. Local adaptation of *A*. *thaliana* populations to abiotic and biotic stresses have been widely investigated and demonstrated [2–5]. Accessions collected from locations at the extremes of the abiotic environments occupied by *A*. *thaliana* typically display adaptation to that environment. For example, accessions from locations with extreme winter temperatures exhibit low temperature tolerance [2] and alleles conferring salt tolerance are significantly more frequent in *A*. *thaliana* populations situated in marine coastal environments or near unusually saline soils [3].

Adaptation of *A*. *thaliana* to heavy metals was previously described for accessions collected from Berkeley, CA, Santa Clara, CA [6, 7] and Limeport, PA [6]. The original reports indicated that Santa Clara and Limeport were collected from locations with extreme soil conditions suggesting that these might contain genes conferring adaptation to unusual soil conditions. Santa Clara was collected from a serpentine soils site that is part of the Jasper Ridge preserve with naturally occurring high levels of Ni and a low Ca to Mg ratio [8]. The Jasper Ridge serpentine outcrops are one of the most studied and frequently collected sites for researchers working on serpentine soil adaptation in plants [8, 9]. Many serpentine sites exist in California, and a digital collection of vouchered botanical collections from these sites, as well as all of California, are well characterized in the Jepson database [9]. Limeport was collected from a site in Pennsylvania with high Zn concentrations (http://www.arabidopsis.org) as a result of human mining activity and it was described as Zn tolerant [6]. Incongruously, the Limeport genome encodes the same low-frequency deletion allele of HMA3 present in the standard laboratory accession Col-0 that is responsible for heavy metal sensitivity in this accession [10, 11]. The Berkeley accession was characterized as Cu resistant in the same manuscript, which describes metal resistance for Santa Clara and Limeport [6]. Berkeley was collected from an unpolluted urban setting in California.

Despite the collection of these three accessions from distant new-world locations with dramatically different habitats, 149 SNP markers with genome-wide distribution did not detect genetic variation between them nor did they group with naturalized new world *A*. *thaliana* [12]. Instead, these accessions were found to be genetically indistinguishable from the Col-0 accession that is derived from central Europe. This suggested that they were repeated depositions of the laboratory strain Col-0 into the strain collection by Arabidopsis researchers. Anastasio et al. [12] added these accessions to their “red list” of accessions likely to be stock duplications.

Reassessment of previous work has often led to new insights. For example, the mechanism of plant branching regulation in flavonoid mutants was reassessed and demonstrated to be due to a previously un-identified MAX pathway mutation, blocking strigolactone biosynthesis, in the *tt3* mutant line exhibiting high branching [13]. Similarly, growth suppression by loss of function mutations in the gene encoding the vacuolar pyrophosphatase, *AVP1*, had been ascribed to altered auxin transport. When this was independently reinvestigated, the growth defects in AVP1 loss-of-function mutants found to be directly due to phosphate balance and primary metabolism [14]. Recently a detailed reassessment of a role for AUXIN BINDING PROTEIN 1 (ABP1) found no evidence supporting *ABP1* function as an auxin receptor [15]. Reassessment is particularly useful when there is disagreement in the literature and new approaches have become available, such as was the case for ABP1, where Cas9-mediated direct mutation of ABP1 could be used to generate many deletions in adult plants to circumvent the criticism that *ABP1* deletion would be embryo lethal [15]. A similar paradox in the literature exists for metal tolerance in *A*. *thaliana*. It was demonstrated that alleles at heavy metal atpase loci affecting sensitivity to heavy metal treatments are present in both Limeport and Col-0 [10, 11]. However, it was reported that Limeport was tolerant to Zn compared to Col-0 [6]. As genetic technology has advanced, genotyping at a genome-wide distribution of SNP positions has added to the finding of no genetic differences between Limeport and Col-0 at 149 SNP positions [12]. In the same study that investigated Limeport, two other heavy metal tolerant lines, Berkeley and Santa Clara, were also described [6]. In the same genotyping study that identified Limeport as a stock duplication of Col-0 with no genetic polymorphisms from the standard laboratory accession, Berkeley and Santa Clara were also found to have no genetic differences distinguishing them from Col-0 [12]. The possibility exists, perhaps remote, that these lines are genetically distinct at positions not surveyed in the 149 SNP study that confer heavy metal tolerance. We now can readily sequence the whole genomes of these lines to clarify the relationships between these reportedly metal tolerant accessions and the worldwide diversity of *A*. *thaliana*.

We began a study of natural variation in heavy metal tolerance, due to our interest in identifying within species variation in this aspect of serpentine adaptation in the genetic model *A*. *thaliana*. To this end we carefully described the metal tolerance of Limeport, Santa Clara and Berkeley and characterized the genetic differences between them by whole genome resequencing. All deposited stocks for each of the accessions were obtained from the Arabidopsis Biological Resource Center (ABRC). All stocks were grown on multiple concentrations of Zn, Cu, or Ni and growth was assessed at multiple days after germination in multiple independent grow outs with multiple technical replicates for each. None of these accessions display any resistance to any concentrations of the toxic heavy metals tested when compared to Col-0. Whole genome resequencing did not uncover genetic variation missed by the 149 SNPs used previously. Thus, detailed phenotypic and genotyping characterization of these indicated that all three accessions are certainly re-deposited derivatives of the standard laboratory accession Col-0. No vouchered botanical collections of Arabidopsis were found from the Jasper Ridge serpentine. Furthermore, no additional evidence of any accessions with serpentine adaptation was found by comparing the Jepson database of botanical collections to serpentine soil distributions. We propose that these previously identified metal-adapted accessions do not provide any evidence of heavy metal adaptation in *A*. *thaliana* and that further study of these materials will not provide insight into the mechanisms of serpentine adaptation or edaphic selection at mine sites in plants.

## Materials and Methods

### Plant Material

*A*. *thaliana* lines used are: Columbia-0 (Col-0) from Lehle seed (Round Rock, TX); Limeport (CS28464; CS8070), Berkeley (CS28067; CS8068), and Santa Clara (CS28722; CS8069) from TAIR Stock Center.

To have similar experimental condition to Murphy et al. (1995), Arabidopsis seeds sown on plates with-half strength Murashige and Skoog medium (Murashige and Skoog, 1962; Phytotechlab, Shawnee Mission, KS; catalog no. M404) containing 0.7% agar (Sigma-Aldrich, St. Louis, MO; catalog no. A1296) in the presence or absence of different concentration of nickel (II) nitrate (Acros Organics, Morris Plain, NJ; catalog no. AC223155000), zinc (II) nitrate (J.T Baker, Phillipsburg, NY; catalog no. 4344), or copper (II) chloride (Sigma-Aldrich, St. Louis, MO; catalog no. 467847). Seeds were stratified on the plates at 4°C for 72h. Seeds were germinated and plants grown in vertical orientation on light racks in an environmentally controlled room (24°C/20°C, 12h/12h light/dark). Each plate contained five seeds each of two accessions. The accessions planted per plate were distributed randomly and each accession-treatment combination was replicated five independent times. Comparisons between accessions and treatments were done by ANOVA using Tukey (P<0.05). The connecting letters reports summarizing the Tukey test for each metal-day combination are available in the supplemented data section (S1–S9 Tables). Botanical collection data from the Jepson Interchange database (http://ucjeps.berkeley.edu/interchange/) and serpentine soils map data were overlaid using the R statistical package by Brian Anacker at the University of California, Davis.

### DNA extraction and next-generation sequencing reads mapping

For DNA extraction, *A*. *thaliana* lines were grown in the green house for several weeks and leaves were collected and frozen in liquid nitrogen. Frozen leaves were used to extract DNA for genomic sequencing using the CTAB method [16]. Illumina libraries (v4; Illumina San Diego, CA) were constructed from DNA extracted from each Arabidopsis accession. Barcoded libraries were pooled on a single lane and 275,794,696 100bp paired-end sequences were generated. To call SNPs, reads corresponding to each library were separated and the data from each accession aligned to the latest version of the *A*. *thaliana* reference genome (obtained from http://www.phytozome.net version 9.1) [17]. Alignments were carried out using the BWA short reads aligner program (version 0.62) [18]. To expedite NGS reads mapping, we first generated the suffix array co-ordinates for each sequenced read using the BWA aln command with parameter option: “–t 8”. The suffix array co-ordinates enabled the generation of sequence alignments to the A. thaliana reference genome, using the BWA sampe command with the parameters: -P -r "@RG\tID: SAMPLEID\tSM: SAMPLENAME\tPL:Illumina". The output alignments generated in the SAM NGS alignment format, by the BWA program is converted to the compressed BAM NGS alignment format using the view command in the SAMtools package (version 0.1.18) [19], with parameters: “-b -h –S”. The BAM alignment files were then sorted and indexed using the SAMtools commands sort and index respectively, and with default parameters.

SNP calling was performed using a combination of the SAMtools mpileup comand and the BCFTools view program. The parameters for mpileup were “-B -Q 20 -P Illumina -C50 –uf”. The output of the mpileup program was redirected from the UNIX standard output device to the BCFTools view program, using the standard UNIX/Linux pipe tool. The selected parameters for the BCFTool view command were “-vcg”. The varFilter command in the SAMtools vcfutils.pl utility script (parameter option: “-D100”) was used to remove SNPs derived from repetitive origins. This command also filtered SNPs with quality score less than 10 and within three bases of any gaps detected in the alignment. The UNIX grep utility program (with parameter: –v “INDEL”) was then used to parse the output variant call file, to remove all predicted insertion/deletion (INDEL) variants.

The SnpSift filter command in the SnpEff software suite (version 3.1) [20] was used to filter the remaining SNPs in a stepwise manner. All SNPs with quality scores less than 20 and non-homozygous SNP positions were eliminated using the command string "(QUAL> = 20)& (isHom(GEN[0])) & (isVariant(GEN[0]))". Lastly, only SNP identified on reads from both strands of the genome were retained using the SnpSift filter command string "(((DP4[0] = 0)& (DP4[1] = 0)) & ((DP4[2]>0)& (DP4[3]> 0)))". The resultant file represents the high quality homozygous polymorphisms present in each sequence data set for each accession.

### SNP Effect Annotation

To annotate consequences of each SNP on gene coding capacity, we used the SnpEff program with parameter options: “eff –c”, and the pre-built “athalianaTair10” SnpEff database for the *A*. *thaliana* genome (version 10), to compute the SNP impact. For SNPs overlapping the open reading frames of genes, we appended the gene function description, using the gene annotation file for *A*. *thaliana*, which we obtained from the Phytozome website (version 9.1).

To compare SNPs called in the various *A*. *thaliana* accessions, we used the BEDTools software package (version 2.17.0) [21]. To detect common (overlapping) SNPs for a pair of Arabidopsis accessions, we used the BEDTools intersect command with parameter “-wa”. Accession-specific SNPs were detected using the BEDTools subtract command with parameter option “-A”. To facilitate the comparison of the subset of SNPs in the coding regions of the genome, we used the BEDTools intersect command with parameter option “-wa” to obtain the SNPs overlapping the latest version of the Arabidopsis reference genome annotation (.gff3) file.

## Results

To ensure that the biological materials were as similar to each other as possible, all accessions (see Materials and Methods) were grown in the same greenhouse and fresh mature seeds collected by hand. For the three reportedly metal tolerant Arabidopsis accessions (Berkeley, Santa Clara, and Limeport), we ordered all available depositions of the stocks. Seeds were desiccated and stored in microcentrifuge tubes with a hole punched in the top to maintain high seed quality and low moisture levels. Thus, all genotypes had similar seed qualities and were at similar seed ages to minimize the impact of differences in early seedling establishment on plates from confounding the analysis of metals resistance or growth rates. In addition, this produced enough seed from each accession obtained from the stock center to carry out all treatments on the same batch of seed.

### None of the tested accessions, including Santa Clara, are Ni resistant

Santa Clara was originally described as collected from serpentine soil in Santa Clara County, CA (http://www.arabidopsis.org). This accession has been described as a Ni tolerant ecotype of *A*. *thaliana* [6, 7]. However, Santa Clara did not show any differences with Col-0 in comparative genomic analysis using 149 SNP markers [12].

**Fig 1:**
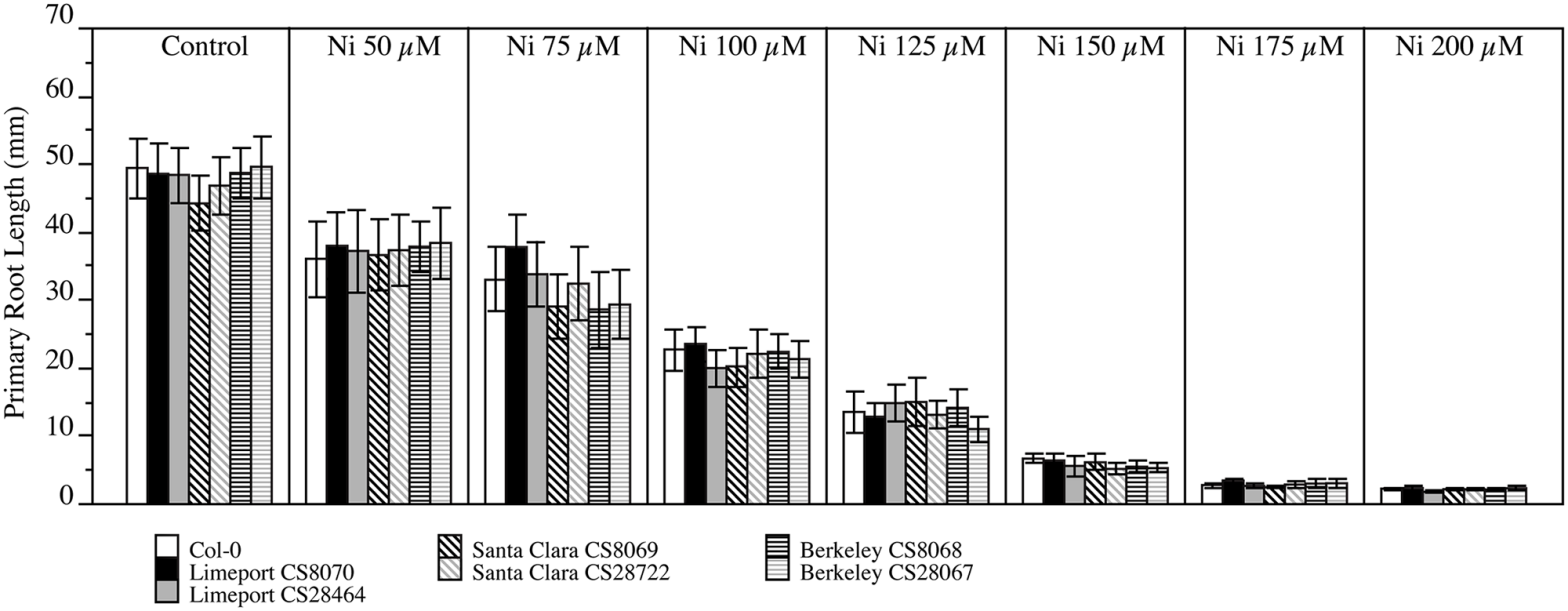
No accessions display differential growth of roots during Nickel treatment. Col-0, Limeport CS8070, Limeport CS28464, Santa Clara CS8069, Santa Clara CS28722, Berkeley CS28067 and Berkeley CS8068 were germinated and grown on solidified one-half Murashige and Skoog supplemented with Ni(NO_3_)_2_ at the indicated concentrations. After 15 days primary root length was measured for each treatment. Data represent the mean (N = 5; ± SE). Comparisons between accessions were done by ANOVA using Tukey (P<0.05).

To confirm the Ni tolerance of Santa Clara [6, 7], both Santa Clara stocks (CS28722 and CS8069) were obtained from the stock center and seed produced. The collected seeds were grown on agar plates supplemented with a range of Ni concentrations. The reportedly Ni-sensitive accessions Limeport, Berkeley and Col-0 [6, 7] were grown in the same conditions as control genotypes. Primary root measurements were taken at 15 days after germination (dag; Fig 1) as well as 10 dag and 20 dag (S1 Fig).

Root growth at a range of Ni concentrations was measured to ensure that we observed plants at physiologically relevant Ni toxicities. At concentrations above 150 μM, Ni is highly toxic for root growth resulting in inhibition over 90% (Fig 1). At 100 μM Ni and 125 μM Ni growth inhibition for all the accessions tested was between 50% and 70%, respectively (Fig 1). At 50 μM Ni and 75 μM Ni, root growth inhibition was approximately 20% and 30%, respectively (Fig 1). Santa Clara, Col-0, Berkeley and Limeport did not show any significant differences in primary root length under any concentration of Ni nor in control conditions. Thus, whether roots were 90% stunted or only 20% shorter than controls none of these accessions were discernably resistant, or differentially sensitive to this plant toxin relative to Col-0 controls. This indicates that Ni has the same inhibition effect in all the accession tested, and that Santa Clara is not Ni resistant.

### None of the tested accessions, including Limeport, are Zn resistant

**Fig 2:**
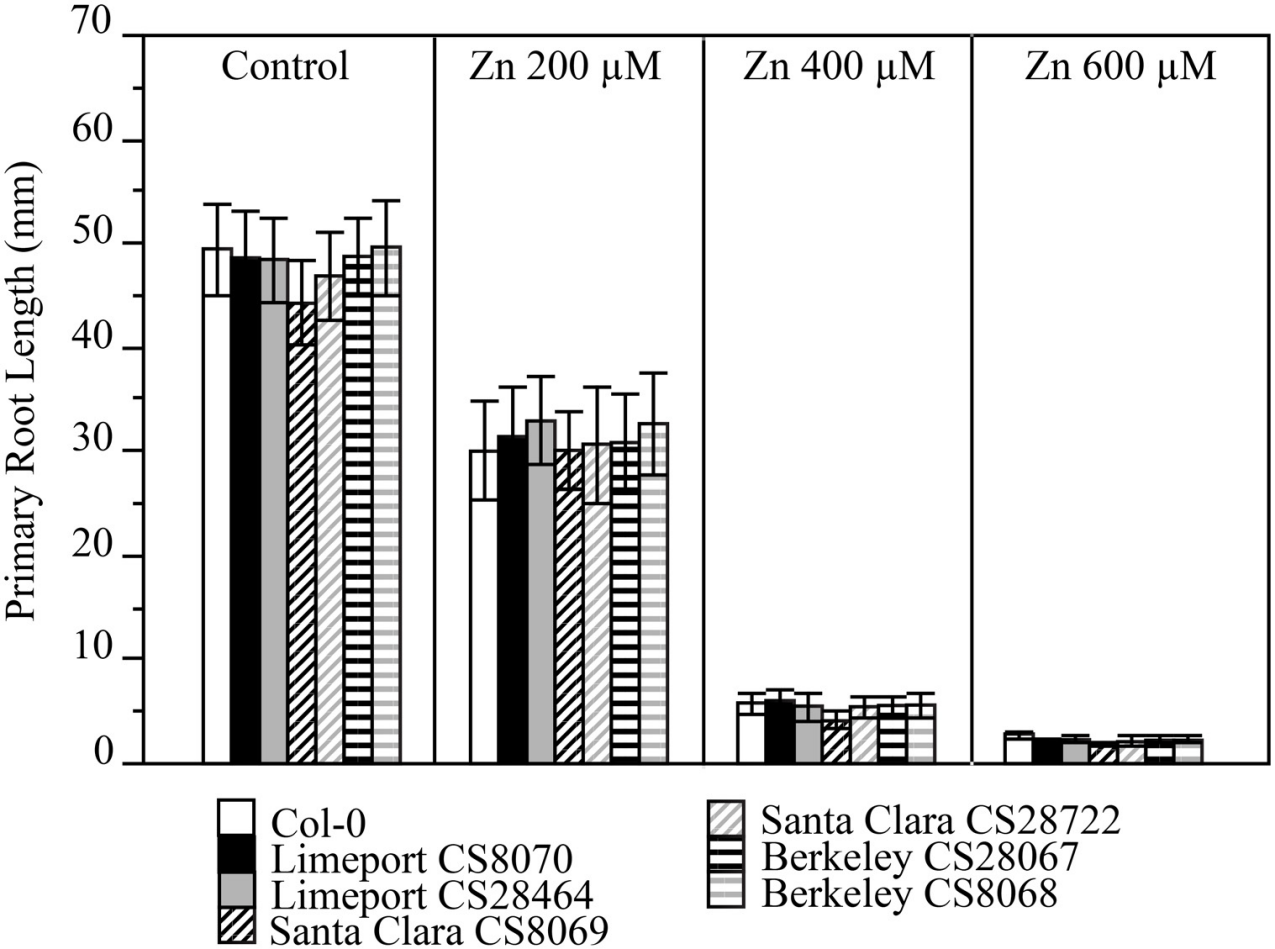
No accessions display differential growth of roots during Zinc treatment. Col-0, Limeport CS8070, Limeport CS28464, Santa Clara CS8069, Santa Clara CS28722, Berkeley CS28067 and Berkeley CS8068 were germinated and grown on solidified one-half Murashige and Skoog supplemented with Zn(NO_3_)_2_ at the indicated concentrations. After 15 days primary root length was measured for each treatment. Data represent the mean (N = 5; ± SE). Comparisons between accessions were done by ANOVA using Tukey (P<0.05).

The Limeport accession was collected from soil within a Zn mining site located in Friedensville, PA (http://www.arabidopsis.org) and was described as Zn resistant in the study that also reports the collection [6]. However, Limeport was genetically similar to the Zn sensitive accession Col-0 when tested at 149 SNPs [12] and contains the same stop codon in HMA3 associated with heavy metal sensitivity as Col-0 [10]. To reassess Zn tolerance of the Limeport accession we acquired both Limeport stocks (CS28464 and CS8070) available in the stock center. Mature seeds were sown on agar plates supplemented with a variety of concentrations of Zn. The reportedly Zn-sensitive Santa Clara, Berkeley and Col-0 [6] accessions were grown in parallel as controls. Primary root lengths were measured at 15 dag (Fig 2) as well as 10 dag and 20 dag (S2 Fig). No significant differences were observed between Limeport, Santa Clara, Berkeley and Col-0 under any of the different concentrations of Zn or control conditions for any of the durations of growth tested. For all the accessions tested, we observed that 200 μM Zn induced ~ 40% root growth inhibition and Zn concentrations over 400 μM were extremely toxic causing root growth inhibition over 90% (Fig 2). We conclude Limeport is not Zn resistant despite reported collection from a Zn mining site.

### None of the tested accessions including, Berkeley and Limeport, are Cu resistant

**Fig 3:**
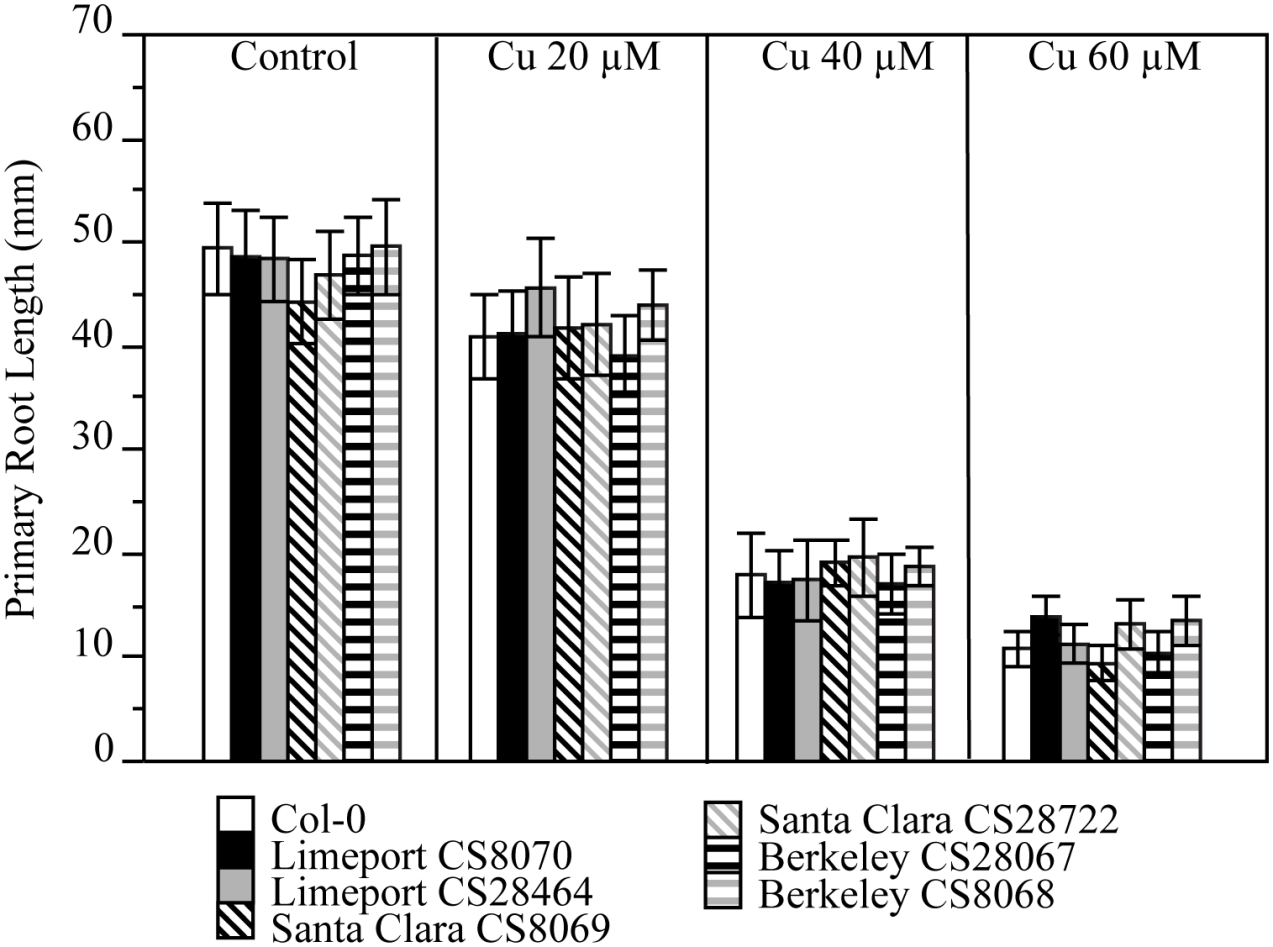
No accessions display differential growth of roots during Copper treatment. Col-0, Limeport CS8070, Limeport CS28464, Santa Clara CS8069, Santa Clara CS28722, Berkeley CS28067 and Berkeley CS8068 were germinated and grown on solidified one-half Murashige and Skoog supplemented with the indicated concentrations of CuCl_2_. After 15 days primary root length was measured for each treatment. Data represent the mean (N = 5; ± SE). Comparisons between accessions were done by ANOVA using Tukey (P<0.05).

The Berkeley accession was collected near the UC Berkeley library in CA (http://www.arabidopsis.org) and was described as Cu resistant in the study that also describe their collection [6]. Like Limeport and Santa Clara, Berkeley was reported to be genetically similar to the Cu sensitive Col-0 at 149 SNP positions [12]. To check if Berkeley is Cu resistant, both Berkeley stocks (CS28067 and CS8068) available in the stock center were grown as described previously. Fresh seed was planted on agar-solidified media containing a variety of concentrations of Cu. The other three accessions Santa Clara, Col-0 and Limeport were grown in the same Cu toxic conditions as controls. Primary root lengths were measured at 15 dag (Fig 3) as well as 10 and 20 dag (S3 Fig). No discernable differences in root length could be detected between Berkeley, Santa Clara, Limeport and Col-0 at any Cu concentration for any duration of growth tested. Again, a physiologically relevant range of Cu toxicities were achieved in these experiments. We observed that 20 μM Cu caused ~ 20% root growth inhibition while concentrations over 40 μM Cu were highly toxic inhibiting root growth by ~60% (Fig 3). Thus, Berkeley was Cu sensitive as Limeport, Santa Clara and Col-0.

### Whole genome resequencing confirms that Limeport, Santa Clara, Berkeley and Col-0 are the same accessions

It was previously showed that Santa Clara, Berkeley, Limeport and Col-0 were genetically similar using 149 SNP markers [12]. Given that thousands of accessions were genotyped it is possible that these similarities are spurious. It is also possible that mutations of adaptive significance, not included among the 149 polymorphic positions are present in these lines. The genomes of each accession were resequenced to distinguish between a spurious similarity at 149 positions, and genetic identity between Col-0, Santa Clara, Limeport and Berkeley. Alignment of reads to the *A*. *thaliana* Col-0 reference genome permitted calling of SNPs relative to Col-0. SNPs were compared between each accession pair (Table 1). Any SNPs present in two accessions were flagged as “Common SNPs”. By comparing the called SNPs in Santa Clara, Limeport and Berkeley to the SNPs called in the resequenced Col-0 we were able to identify all putative sites of divergence for these accessions, and distinguish them from errors in the reference genome or error prone positions.

**Table 1:** Comparison of high quality SNPs between accessions.

Ecotype 1 (E1)	SNPs in E1	Ecotype 2 (E2)	SNPs in E2	SNPs in E1 and E2	SNPs only in E1	SNPs only in E2^a^
Limeport CC28464	858	Limeport CC8070	855	782	76	73
Berkeley CC28067	881	Berkeley CC8068	878	802	79	76
Santa Clara CC28722	885	Santa Clara CC8069	870	794	91	76
Santa Clara CC8069	870	Limeport CC8070	855	774	96	81
Santa Clara CC28722	885	Limeport CC8070	855	774	111	81
Berkeley CC8068	878	Limeport CC8070	855	771	107	84
Berkeley CC28067	881	Limeport CC8070	855	770	111	85
Col-0	892	Limeport CC8070	855	767	125	**88**
Santa Clara CC8069	870	Limeport CC28464	858	766	104	92
Santa Clara CC28722	885	Limeport CC28464	858	768	117	90
Berkeley CC8068	878	Limeport CC28464	858	771	107	87
Berkeley CC28067	881	Limeport CC28464	858	769	112	89
Col-0	892	Limeport CC28464	858	764	128	**94**
Berkeley CC8068	878	Santa Clara CC8069	870	792	86	78
Berkeley CC28067	881	Santa Clara CC8069	870	778	103	92
Col-0	892	Santa Clara CC8069	870	778	114	**92**
Berkeley CC8068	878	Santa Clara CC28722	885	785	93	100
Berkeley CC28067	881	Santa Clara CC28722	885	791	90	94
Col-0	892	Santa Clara CC28722	885	784	108	**101**
Col-0	892	Berkeley CC8068	878	785	107	**93**
Col-0	892	Berkeley CC28067	881	773	119	**108**

^a^ The numbers in bold are the high quality SNPs that are not also present in the Col-0 sequence

We identified between 855 and 892 SNPs between each accession and the digital reference genome. These are orders of magnitude less variation than identified between accessions [22–25]. When comparing each line pair-wise, we observed that the numbers of shared SNPs were similar whether we compared between the two stock numbers for the same accession and/or between accessions for Santa Clara, Berkeley or Limeport (Table 1). Surprisingly, the sequence of Col-0 was similarly different from the reference genome and the same SNPs were often present in Col-0 as in the other accessions. This indicated that the source of variation within Limeport, Santa Clara and Berkeley was similar to that in Col-0 and likely within the margins of error for Illumina sequencing. This strongly confirms the results of Anastasio et al. (2011) that Col-0, Santa Clara, Limeport and Berkeley are genetically identical.

**Fig 4:**
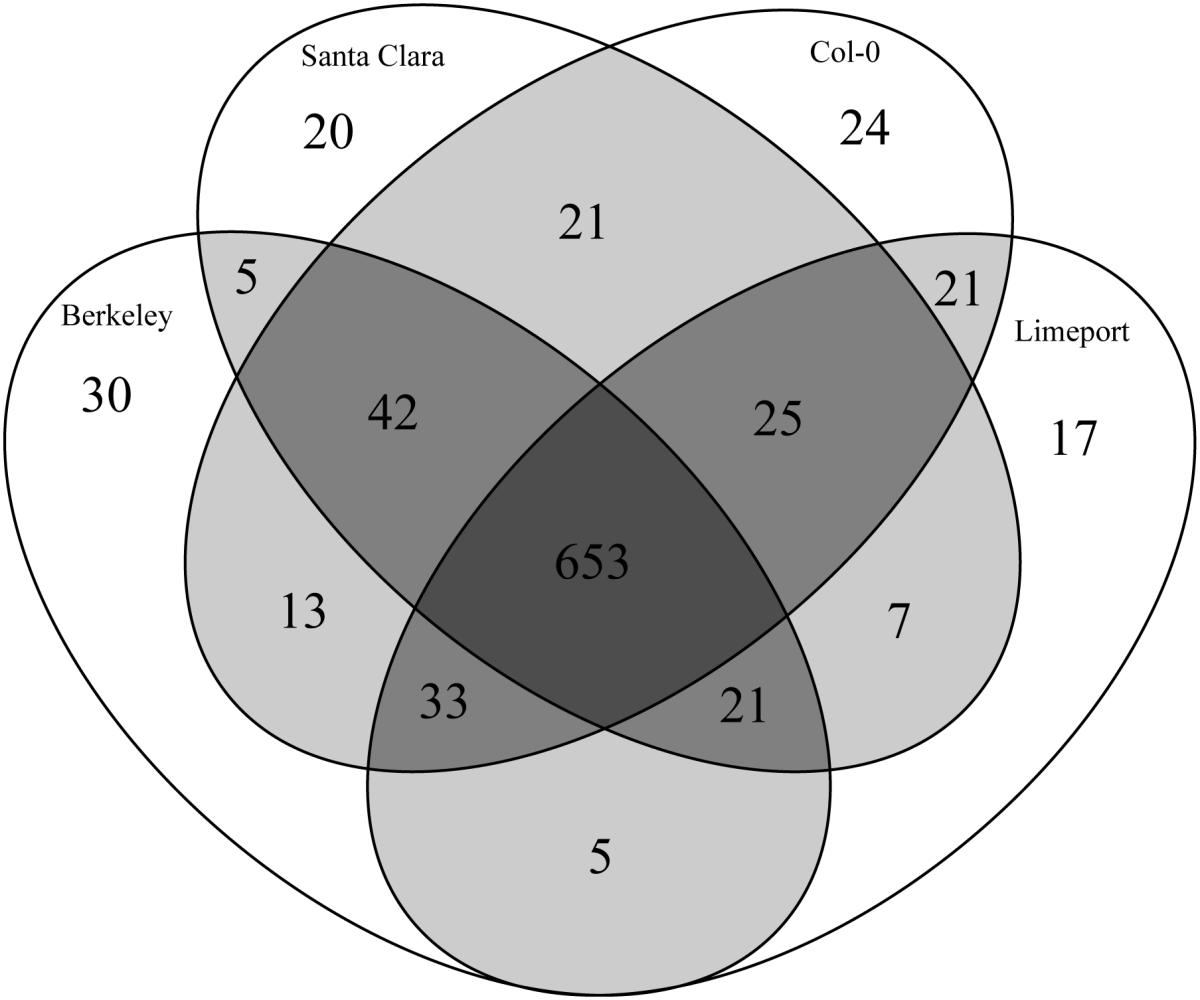
Venn diagram of common SNPs between Col-0, Limeport, Santa Clara and Berkeley. The diagram was constructed using the common SNPs between Limeport CS8070 and Limeport CS28464; Santa Clara CS8069 and Santa Clara CS28722; Berkeley CS28067 and Berkeley CS8068.

To obtain better estimates of the base substitution rate between lines, we identified all SNPs present in both stocks of an accession and not contained in Col-0. Fig 4 summarizes the shared alleles between the four genotypes as a Venn diagram, and Table 2 contains SNP counts for the most informative categories. If these lines were resistant to different metals, the alleles responsible for these differences would have to be unshared, so we also determined the SNPs present in both sequencing experiments for a genotype and absent in any other accessions. These are referred to as the unique SNPs for each accession (Table 2). These comparisons indicated that the variation between Santa Clara, Limeport, Berkeley and Col-0 is low (Table 2). Of these, 653 are shared between all accessions (Fig 4), 20 times more than the number of unique SNPs per accessions (Table 2). Further, the number of common SNPs private to any three or two-way comparison is much lower than the common SNPs shared among all the accessions tested. Given that Col-0 itself exhibits 24 SNPs private to that line this indicates that the variability between Limeport, Santa Clara and Berkeley is likely to be insignificant and close to the error rate of sequencing.

**Table 2:** Number of common and unique SNPs for Col-0, Limeport, Santa Clara and Berkeley.

	Reproducible SNPs	SNPs shared with Col-0	SNPs distinct from Col-0	SNPs unique to line
Limeport	782	732 (93.6%)	50 (6.4%)	17 (2.2%)
Santa Clara	794	741 (93.3%)	53 (6.7%)	20 (2.5%)
Berkeley	802	741 (92.4%)	61 (7.6%)	30 (3.7%)
Col-0	892	na	na	24 (2.7%)

### Nonsynonymous variation is highly unlikely to affect metal tolerance in Santa Clara, Limeport and Berkeley genomes

We showed that Berkeley, Santa Clara, Limeport and Col-0 are nearly identical by whole genome sequencing and suffer similar inhibition of root growth in response to Ni (Fig 1 and S1 Fig), Zn (Fig 2 and S2 Fig) and Cu (Fig 3 and S3 Fig). Since only one sequence change is needed to provide metal resistance we scanned the putative changes for non-synonymous changes to genes associated with antioxidant mechanisms and metal transport. The unique non-synonymous SNPs for each accession were identified. Only one non-synonymous SNP was unique to Limeport (S10 Table), four to Santa Clara (S11 Table) and two to Berkeley (S12 Table). None of these fell in genes associated with known metal tolerance mechanisms, consistent with the observation of no alterations to metal resistance in any of the tests performed here (Figs 1–3 and S1–S3 Figs).

### Botanical samples of *A*. *thaliana* in California

**Fig 5:**
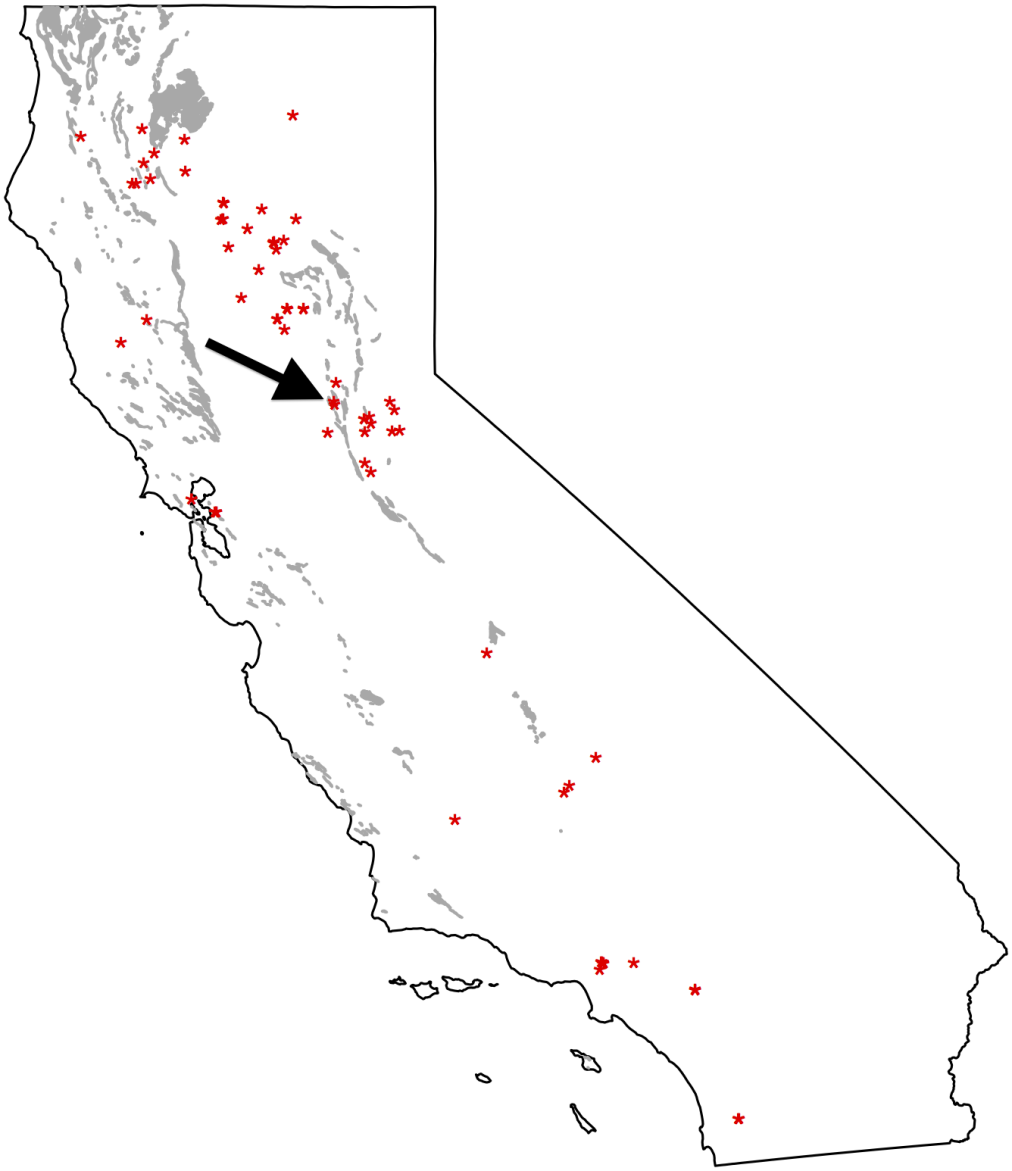
Locations of *A*. *thaliana* collection and serpentine soil presence in California. Each dot on the map represents a different locality. The arrow indicates the only accession within 400 M of known serpentine soils.

Given the general interest in serpentine adaptation, *A*. *thaliana* in general, and the Jasper ridge serpentine in particular, we considered that evidence of *A*. *thaliana* serpentine adaptation might be found using the online Jepson collection http://ucjeps.berkeley.edu/. We expect that *A*. *thaliana* growing in California serpentine sites would be evident in the online Jepson collection. No botanically vouchered Arabidopsis collections made on the Jasper ridge serpentine were present in the Jepson database (Fig 5). Of the hundreds of data based collections of *A*. *thaliana* made in California, only one was taken from within 400 meters of serpentine soil. The one accession closest to a serpentine soils site (http://ucjeps.berkeley.edu/cgi-bin/new_detail.pl?accn_num=RSA781117) was taken from a gravel trail that runs through the known serpentine site at Salmon Falls in El Dorado County in North Central California. Thus, the digitized botanical collections failed to identify any other record of an *A*. *thaliana* population growing on serpentine soil in Santa Clara [6] and provides no evidence for serpentine adaptation for any of the hundreds of *A*. *thaliana* populations recorded in California.

## Discussion

*A*. *thaliana* displays local adaptation to a variety of environments across a wide geographic range [2–5]. The genetic divergence between accessions is correlated with the distance between collection sites. Accessions that grow in close locations are genetically similar, but accessions that grow in distant location are genetically different between each other [12, 26]. It was previously observed, that Santa Clara, Berkeley, Limeport and Col-0 are genetically indistinguishable but collected from distant and distinct habitats [12]. Using whole genome sequencing, we observed negligible variability between Santa Clara, Limeport, Berkeley and Col-0 (Tables 1 and 2, Fig 4).

Santa Clara was described as collected from serpentine soil. This is the only *A*. *thaliana* taken from an extreme soil environment with any indication that it is able to survive normally toxic levels of Ni [6, 7]. In our reanalysis, we failed to detect any greater tolerance to any concentration of Ni than was seen in Col-0 (Fig 1). Furthermore, no genetic variation between Santa Clara and Col-0 was likely to affect metal tolerance under untested conditions (S11 Table). It is important to note that we utilized the Santa Clara accessions available from the Arabidopsis Biological Resource Center, whereas Agrawal et al. [7] cited the Lehle Seeds company as their source for Santa Clara. Limeport was collected from Zn rich mine soil and was characterized as a Zn tolerant genotype [6]. Across Ni, Zn, and Cu doses and treatment times, Limeport responded identically to the Ni, Zn and Cu-sensitive Col-0 demonstrating that Limeport is not Zn tolerant (Fig 2). Further, no genetic variation associated with metal tolerance was found when Limeport was compared to Col-0 (S10 Table). Berkeley was initially described as Cu-tolerant [6], but then later described as Cu sensitive by the same authors [27]. Although, both articles grew Arabidopsis on vertically oriented chromatography paper saturated with Murashige -Skoog medium and supplemented with different metal concentrations, the authors explained that the different response of Berkeley to Cu occurred for “significant differences in the growth assay” [6, 27]. We demonstrated not only that Berkeley is genetically near-identical to Col-0 but also that Berkeley is Ni, Zn, and Cu sensitive just like Col-0 when exposed to increasing toxic concentrations of the metals (Fig 3).

Based on the sequencing data alone, we conclude that Santa Clara, Limeport, Berkeley and Col-0 are the same accessions. The identical physiological behaviors of these lines under different concentrations of toxic metals and control conditions are consistent with this finding. We strongly suggest that these accessions not be used to study natural variation in metal adaption or any other study. *A*. *thaliana* does show differences in Ca/Mg ratio tolerance [28, 29] as well genetically modified lines are tolerance to abiotic stresses including heavy metals [30–33]. Nevertheless, based on collection locations for *A*. *thaliana* present in the Jepson database and their non-alignment with serpentine soil sites, we suggest that *A*. *thaliana* is unlikely to display natural variation across this edaphic range. *A*. *thaliana* is not the right model organism to study genetic adaptation to these unusual and fascinating soils. Detailed mechanistic studies of the myriad serpentine adapted taxa in experimentally tractable genera such as *Aquilegia*, *Mimulus*, *Thlaspi*, *Alyssyum*, or *Noccaea* [34–39] remains the best route to understand the basis of serpentine adaptation in plants.

## Supporting Information

S1 Dataset Dataset containing SNP calls from the accessions sequenced in this study. (ZIP)

S1 Fig No accessions display differential growth of roots during Nickel treatment. Col-0, Limeport CS8070, Limeport CS28464, Santa Clara CS8069, Santa Clara CS28722, Berkeley CS28067 and Berkeley CS8068 were germinated and grown on solidified one-half Murashige and Skoog supplemented with Ni(NO_3_)_2_ at the indicated concentrations. After (A) 10 days and (B) 20 days primary root length was measured for each treatment. Data represent the mean (N = 5; ± SE). Comparisons between accessions were done by ANOVA using Tukey (P<0.05).
(TIF)

S2 Fig No accessions display differential growth of roots during Zinc treatment. Col-0, Limeport CS8070, Limeport CS28464, Santa Clara CS8069, Santa Clara CS28722, Berkeley CS28067 and Berkeley CS8068 were germinated and grown on solidified one-half Murashige and Skoog supplemented with Zn(NO_3_)_2_ at the indicated concentrations. After (A) 10 days and (B) 20 days primary root length was measured for each treatment. Data represent the mean (N = 5; ± SE). Comparisons between accessions were done by ANOVA using Tukey (P<0.05).
(TIF)

S3 Fig No accessions display differential growth of roots during Copper treatment. Col-0, Limeport CS8070, Limeport CS28464, Santa Clara CS8069, Santa Clara CS28722, Berkeley CS28067 and Berkeley CS8068 were germinated and grown on solidified one-half Murashige and Skoog supplemented with the indicated concentrations of CuCl_2_. After (A) 10 days and (B) 20 days primary root length was measured for each treatment. Data represent the mean (N = 5; ± SE). Comparisons between accessions were done by ANOVA using Tukey (P<0.05).
(TIF)

S1 Table Connecting letters report for zinc treatment at day 10. (DOCX)

S2 Table Connecting letters report for zinc treatment at day 15. (DOCX)

S3 Table Connecting letters report for zinc treatment at day 20. (DOCX)

S4 Table Connecting letters report for nickel treatment at day 10. (DOCX)

S5 Table Connecting letters report for nickel treatment at day 15. (DOCX)

S6 Table Connecting letters report for nickel treatment at day 20. (DOCX)

S7 Table Connecting letters report for copper treatment at day 10. (DOCX)

S8 Table Connecting letters report for copper treatment at day 15. (DOCX)

S9 Table Connecting letters report for copper treatment at day 20. (DOCX)

S10 Table All non-synonymous changes unique to the Limeport accessions. (DOCX)

S11 Table All non-synonymous changes unique to the Santa Clara accessions. (DOCX)

S12 Table All non-synonymous changes unique to the Berkeley accessions. (DOCX)

## References

[1] Wixon J. Featured Organism: *Arabidopsis thaliana*. Comparative and Functional Genomics. 2001;2(2):91–8. 10.1002/cfg.75 18628901 PMC2447196

[2] Hannah MA, Wiese D, Freund S, Fiehn O, Heyer AG, Hincha DK. Natural genetic variation of freezing tolerance in Arabidopsis. Plant physiology. 2006;142(1):98–112. 10.1104/pp.106.081141 16844837 PMC1557609

[3] Baxter I, Brazelton JN, Yu D, Huang YS, Lahner B, Yakubova E, et al A coastal cline in sodium accumulation in *Arabidopsis thaliana* is driven by natural variation of the sodium transporter AtHKT1;1. PLoS genetics. 2010;6(11):e1001193–e. 10.1371/journal.pgen.1001193 21085628 PMC2978683

[4] Fournier-Level A, Korte A, Cooper MD, Nordborg M, Schmitt J, Wilczek AM. A map of local adaptation in *Arabidopsis thaliana*. Science. 2011;334(6052):86–9. 10.1126/science.1209271 . 21980109

[5] Hancock AM, Brachi B, Faure N, Horton MW, Jarymowycz LB, Sperone FG, et al Adaptation to climate across the *Arabidopsis thaliana* genome. Science (New York, NY). 2011;334(6052):83–6. 10.1126/science.1209244 21980108

[6] Murphy A, Taiz L. A New Vertical Mesh Transfer Technique for Metal-Tolerance Studies in Arabidopsis (Ecotypic Variation and Copper-Sensitive Mutants). Plant physiology. 1995;108(1):29–38. 12228451 10.1104/pp.108.1.29 PMC157302

[7] Agrawal B, Lakshmanan V, Kaushik S, Bais HP. Natural variation among Arabidopsis accessions reveals malic acid as a key mediator of Nickel (Ni) tolerance. Planta. 2012;236(2):477–89. 10.1007/s00425-012-1621-2 . 22411507

[8] Alexander EB, Coleman RG, Keeler-Wolfe T, Harrison SP. Serpentine geoecology of western North America: geology, soils, and vegetation. New York: New York: Oxford University Press; 2007.

[9] Baldwin BG, Goldman D, Keil DJ, Patterson R, Rosatti TJ. Vascular Plants of California, Thoroughly Revised and Expanded The Jepson Manual. Berkeley, California University of California Press; 2002.

[10] Morel M, Crouzet J, Gravot A, Auroy P, Leonhardt N, Vavasseur A, et al AtHMA3, a P1B-ATPase allowing Cd/Zn/Co/Pb vacuolar storage in Arabidopsis. Plant physiology. 2009;149(2):894–904. 10.1104/pp.108.130294 19036834 PMC2633814

[11] Hussain I, Ur M, Khattak R, Khan F. Analysis of Heavy Metals in Selected Medicinal Plants from Dir, Swat and Peshawar Districts of Khyber Pakhtunkhwa. 2011;33(4):495–8.

[12] Anastasio AE, Platt A, Horton M, Grotewold E, Scholl R, Borevitz JO, et al Source verification of mis-identified *Arabidopsis thaliana* accessions. The Plant journal: for cell and molecular biology. 2011;67(3):554–66. 10.1111/j.1365-313X.2011.04606.x . 21481029

[13] Bennett T, Sieberer T, Willett B, Booker J, Luschnig C, Leyser O. The Arabidopsis MAX pathway controls shoot branching by regulating auxin transport. Current biology: CB. 2006;16(6):553–63. Epub 2006/03/21. 10.1016/j.cub.2006.01.058 . 16546078

[14] Ferjani A, Segami S, Horiguchi G, Muto Y, Maeshima M, Tsukaya H. Keep an eye on PPi: the vacuolar-type H+-pyrophosphatase regulates postgerminative development in Arabidopsis. The Plant cell. 2011;23(8):2895–908. Epub 2011/08/25. 10.1105/tpc.111.085415 ; PubMed Central PMCID: PMCPmc3180799. 21862707 PMC3180799

[15] Gao Y, Zhang Y, Zhang D, Dai X, Estelle M, Zhao Y. Auxin binding protein 1 (ABP1) is not required for either auxin signaling or Arabidopsis development. Proceedings of the National Academy of Sciences. 2015;112(7):2275–80. 10.1073/pnas.1500365112 PMC4343106 25646447

[16] Rogers SO, Bendich AJ. Extraction of DNA from milligram amounts of fresh, herbarium and mummified plant tissues. Plant molecular biology. 1985;5(2):69–76. 10.1007/BF00020088 24306565

[17] Goodstein DM, Shu S, Howson R, Neupane R, Hayes RD, Fazo J, et al Phytozome: a comparative platform for green plant genomics. Nucleic acids research. 2011 10.1093/nar/gkr944 PMC3245001 22110026

[18] Li H, Durbin R. Fast and accurate short read alignment with Burrows-Wheeler transform. Bioinformatics (Oxford, England). 2009;25(14):1754–60. Epub 2009/05/20. 10.1093/bioinformatics/btp324 ; PubMed Central PMCID: PMCPmc2705234. 19451168 PMC2705234

[19] Li H, Handsaker B, Wysoker A, Fennell T, Ruan J, Homer N, et al The Sequence Alignment/Map format and SAMtools. Bioinformatics (Oxford, England). 2009;25(16):2078–9. Epub 2009/06/10. 10.1093/bioinformatics/btp352 ; PubMed Central PMCID: PMCPmc2723002. 19505943 PMC2723002

[20] Cingolani P, Platts A, Wang le L, Coon M, Nguyen T, Wang L, et al A program for annotating and predicting the effects of single nucleotide polymorphisms, SnpEff: SNPs in the genome of *Drosophila melanogaste*r strain w1118; iso-2; iso-3. Fly. 2012;6(2):80–92. Epub 2012/06/26. 10.4161/fly.19695 ; PubMed Central PMCID: PMCPmc3679285. 22728672 PMC3679285

[21] Quinlan AR, Hall IM. BEDTools: a flexible suite of utilities for comparing genomic features. Bioinformatics (Oxford, England). 2010;26(6):841–2. Epub 2010/01/30. 10.1093/bioinformatics/btq033 ; PubMed Central PMCID: PMCPmc2832824. 20110278 PMC2832824

[22] Long Q, Rabanal FA, Meng D, Huber CD, Farlow A, Platzer A, et al Massive genomic variation and strong selection in *Arabidopsis thaliana* lines from Sweden. Nature genetics. 2013;45(8):884–90. 10.1038/ng.2678. Available: http://www.nature.com/ng/journal/v45/n8/abs/ng.2678.html-supplementary-information. 23793030 PMC3755268

[23] Gan X, Stegle O, Behr J, Steffen JG, Drewe P, Hildebrand KL, et al Multiple reference genomes and transcriptomes for *Arabidopsis thaliana*. Nature. 2011;477(7365):419–23. http://www.nature.com/nature/journal/v477/n7365/abs/nature10414.html-supplementary-information. 10.1038/nature10414 21874022 PMC4856438

[24] Cao J, Schneeberger K, Ossowski S, Gunther T, Bender S, Fitz J, et al Whole-genome sequencing of multiple *Arabidopsis thaliana* populations. Nature genetics. 2011;43(10):956–63. Epub 2011/08/30. 10.1038/ng.911 . 21874002

[25] Schneeberger K, Ossowski S, Ott F, Klein JD, Wang X, Lanz C, et al Reference-guided assembly of four diverse *Arabidopsis thaliana* genomes. Proceedings of the National Academy of Sciences of the United States of America. 2011;108(25):10249–54. Epub 2011/06/08. 10.1073/pnas.1107739108 ; PubMed Central PMCID: PMCPmc3121819. 21646520 PMC3121819

[26] Platt A, Horton M, Huang YS, Li Y, Anastasio AE, Mulyati NW, et al The scale of population structure in *Arabidopsis thaliana*. PLoS genetics. 2010;6(2):e1000843 10.1371/journal.pgen.1000843 20169178 PMC2820523

[27] Murphy A, Taiz L. Correlation between potassium efflux and copper sensitivity in 10 Arabidopsis ecotypes. New Phytologist. 1997;136(2):211–22. 10.1046/j.1469-8137.1997.00738.x

[28] Bradshaw HDJ. Mutations in CAX1 produce phenotypes characteristic of plants tolerant to serpentine soils. The New phytologist. 2005;167(1):81–8. Epub 2005/06/14. 10.1111/j.1469-8137.2005.01408.x . 15948832

[29] Lenz H, Dombinov V, Dreistein J, Reinhard MR, Gebert M, Knoop V. Magnesium deficiency phenotypes upon multiple knockout of *Arabidopsis thaliana* MRS2 clade B genes can be ameliorated by concomitantly reduced calcium supply. Plant & cell physiology. 2013;54(7):1118–31. Epub 2013/05/01. 10.1093/pcp/pct062 . 23628997

[30] Howden R, Andersen CR, Goldsbrough PB, Cobbett CS. A cadmium-sensitive, glutathione-deficient mutant of *Arabidopsis thaliana*. Plant physiology. 1995;107(4):1067–73. 7770518 10.1104/pp.107.4.1067 PMC157238

[31] Freeman JL, Garcia D, Kim D, Hopf A, Salt DE. Constitutively elevated salicylic acid signals glutathione-mediated nickel tolerance in *Thlaspi* nickel hyperaccumulators. Plant physiology. 2005;137(3):1082–91. 10.1104/pp.104.055293 15734913 PMC1065408

[32] Nishida S, Tsuzuki C, Kato A, Aisu A, Yoshida J, Mizuno T. AtIRT1, the primary iron uptake transporter in the root, mediates excess nickel accumulation in *Arabidopsis thaliana*. Plant & cell physiology. 2011;52(8):1433–42. 10.1093/pcp/pcr089 21742768

[33] Hanikenne M, Talke IN, Haydon MJ, Lanz C, Nolte A, Motte P, et al Evolution of metal hyperaccumulation required cis-regulatory changes and triplication of HMA4. Nature. 2008;453(7193):391–5. 10.1038/nature06877 . 18425111

[34] Ghasemi R, Ghaderian SM, Krämer U. Interference of nickel with copper and iron homeostasis contributes to metal toxicity symptoms in the nickel hyperaccumulator plant *Alyssum inflatum*. The New phytologist. 2009;184(3):566–80. 10.1111/j.1469-8137.2009.02993.x . 19691676

[35] Lochlainn OS, Bowen HC, Fray RG, Hammond JP, King GJ, White PJ, et al Tandem quadruplication of HMA4 in the zinc (Zn) and cadmium (Cd) hyperaccumulator *Noccaea caerulescens*. PloS one. 2011;6(3):e17814 10.1371/journal.pone.0017814 21423774 PMC3053397

[36] Hammond JP, Bowen HC, White PJ, Mills V, Pyke KA, Baker AJ, et al A comparison of the *Thlaspi caerulescens* and *Thlaspi arvense* shoot transcriptomes. The New phytologist. 2006;170(2):239–60. 10.1111/j.1469-8137.2006.01662.x . 16608451

[37] Krämer U. Metal hyperaccumulation in plants. Annual review of plant biology. 2010;61:517–34. 10.1146/annurev-arplant-042809-112156 . 20192749

[38] Callahan DL, Kolev SD, O'Hair RA, Salt DE, Baker AJ. Relationships of nicotianamine and other amino acids with nickel, zinc and iron in *Thlaspi* hyperaccumulators. The New phytologist. 2007;176(4):836–48. 10.1111/j.1469-8137.2007.02216.x . 17897323

[39] Pollard AJ, Reeves RD, Baker AJ. Facultative hyperaccumulation of heavy metals and metalloids. Plant Science. 2014;217–218:8–17. 10.1016/j.plantsci.2013.11.011 . 24467891

