# Supplementary figures and images for: Re-Evaluation of Reportedly Metal Tolerant Arabidopsis thaliana Accessions
Source: PLoS One. 2016 Jul 28;11(7):e0130679. doi: 10.1371/journal.pone.0130679 (PMC4965157; doi:10.1371/journal.pone.0130679)

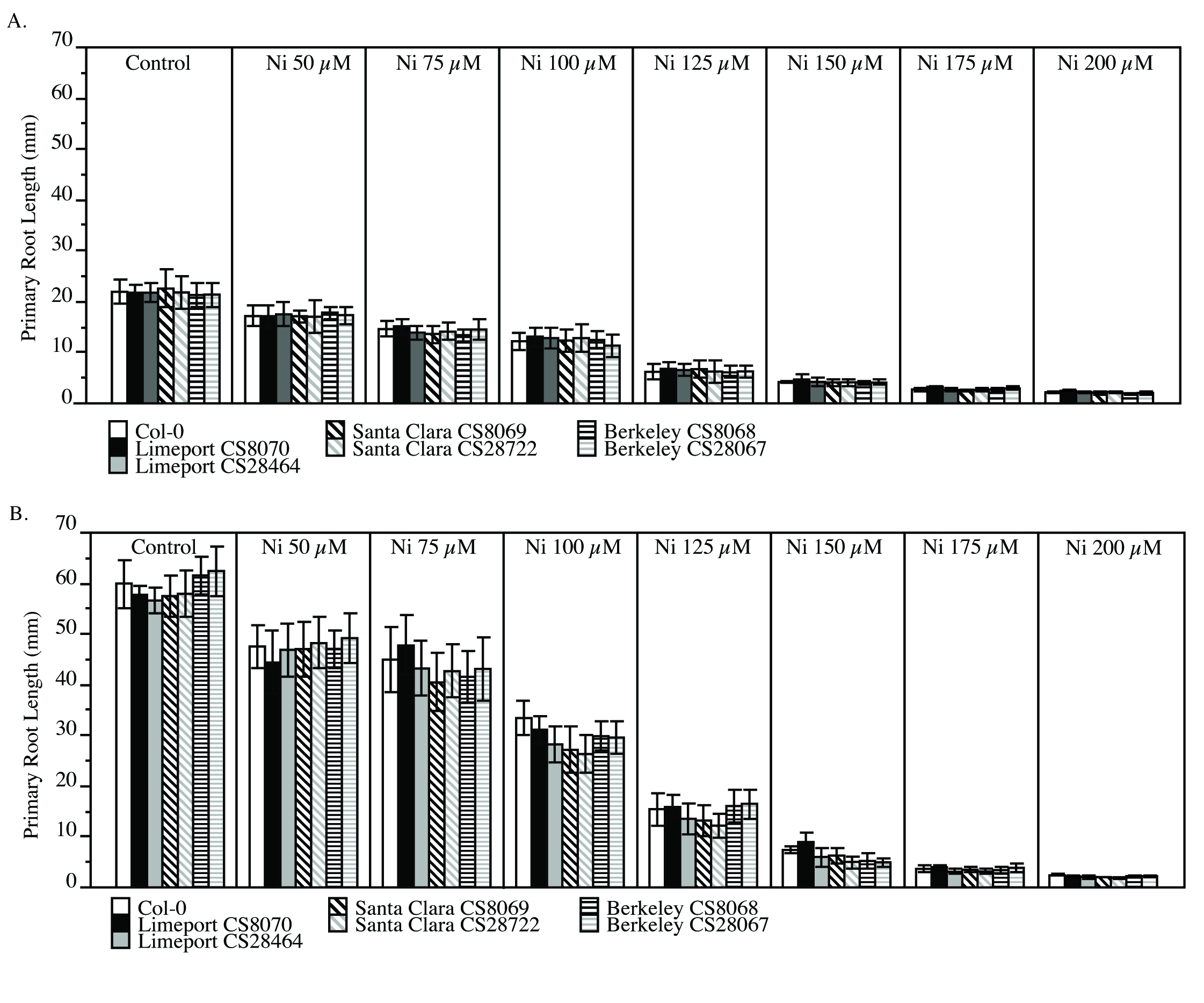

Supplement: S1 Fig — Col-0, Limeport CS8070, Limeport CS28464, Santa Clara CS8069, Santa Clara CS28722, Berkeley CS28067 and Berkeley CS8068 were germinated and grown on solidified one-half Murashige and Skoog supplemented with Ni(NO3)2 at the indicated concentrations. After (A) 10 days and (B) 20 days primary root length was measured for each treatment. Data represent the mean (N = 5; ± SE). Comparisons between accessions were done by ANOVA using Tukey (P<0.05). (TIF) [file pone.0130679.s002.tif]

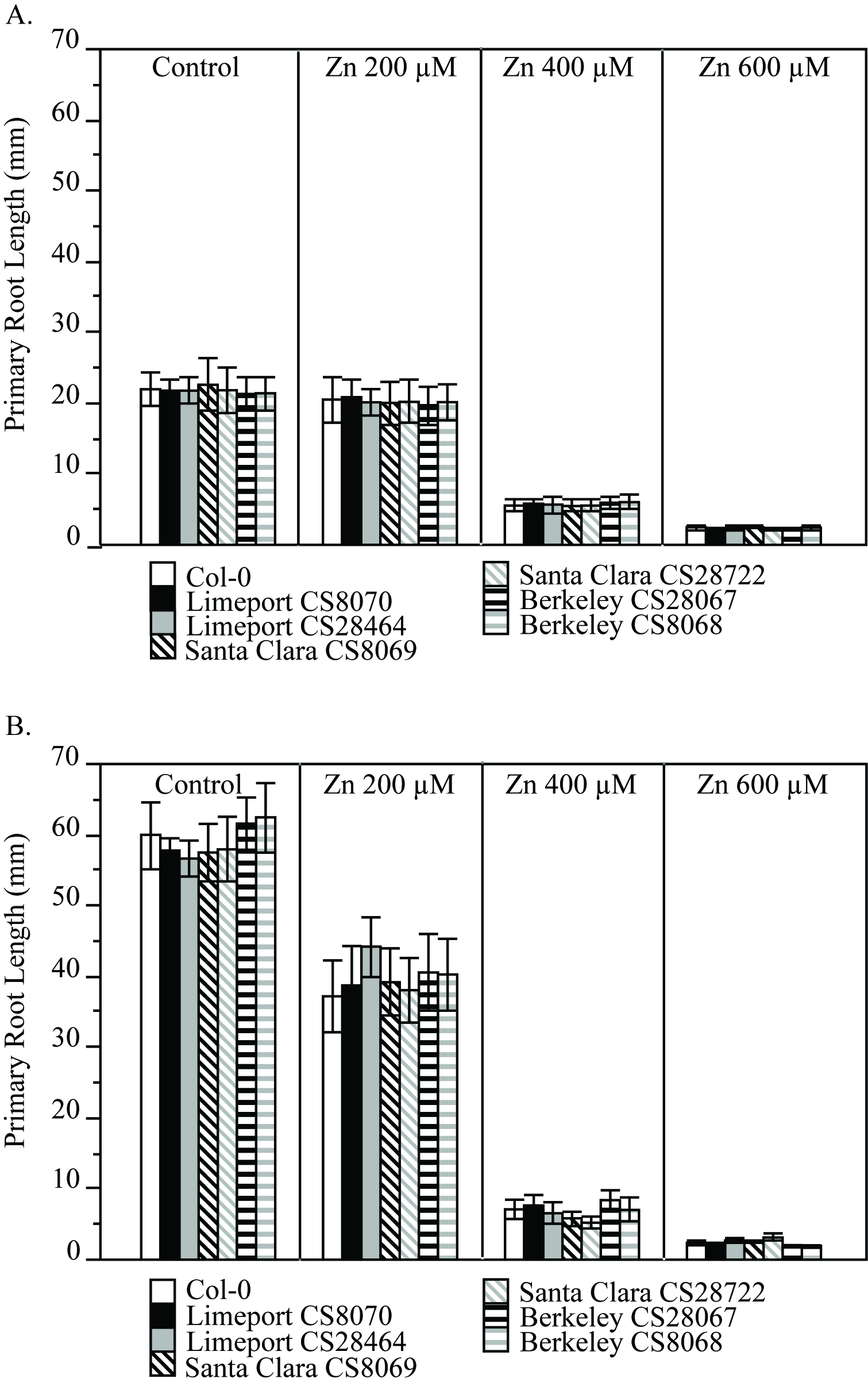

Supplement: S2 Fig — Col-0, Limeport CS8070, Limeport CS28464, Santa Clara CS8069, Santa Clara CS28722, Berkeley CS28067 and Berkeley CS8068 were germinated and grown on solidified one-half Murashige and Skoog supplemented with Zn(NO3)2 at the indicated concentrations. After (A) 10 days and (B) 20 days primary root length was measured for each treatment. Data represent the mean (N = 5; ± SE). Comparisons between accessions were done by ANOVA using Tukey (P<0.05). (TIF) [file pone.0130679.s003.tif]

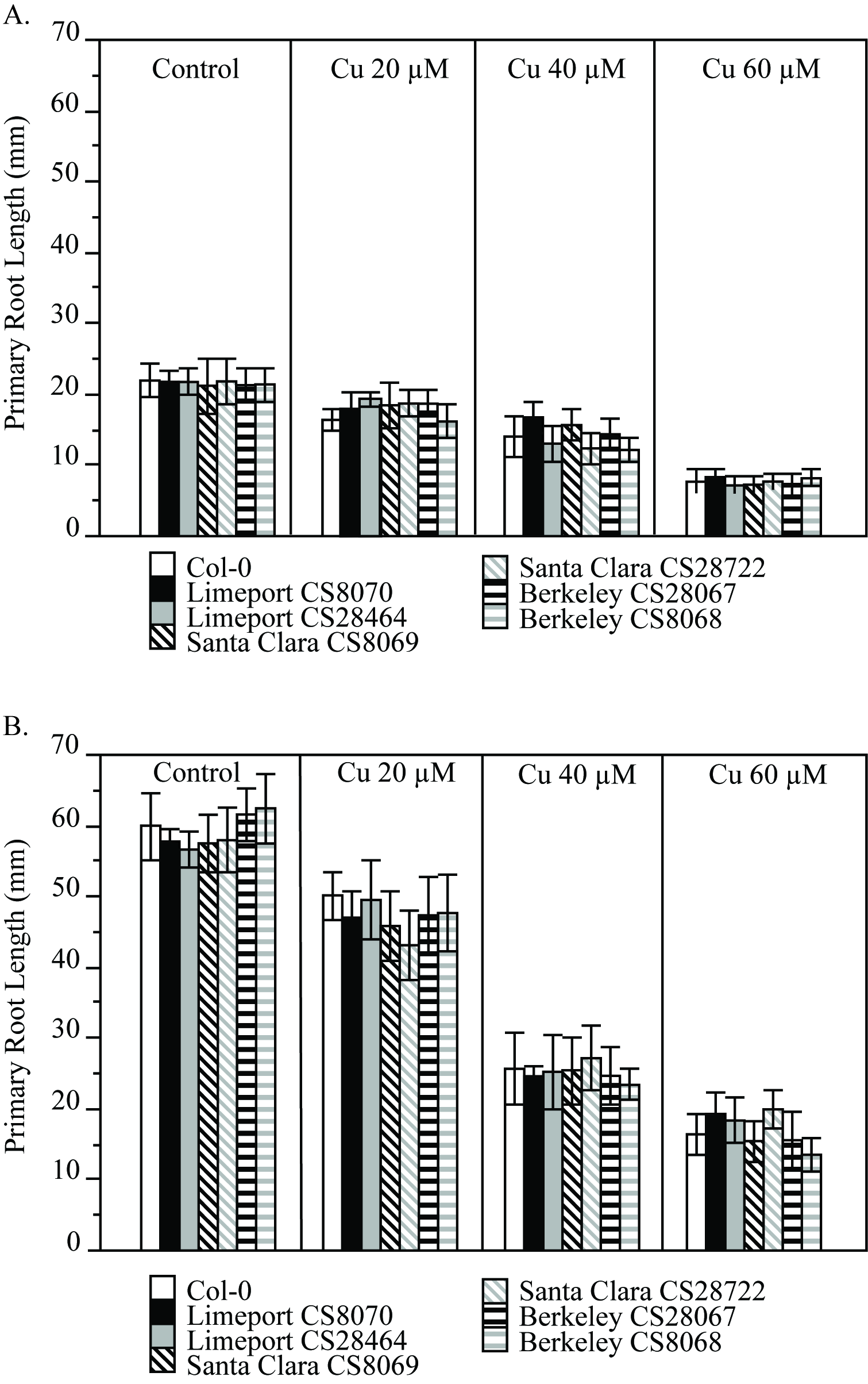

Supplement: S3 Fig — Col-0, Limeport CS8070, Limeport CS28464, Santa Clara CS8069, Santa Clara CS28722, Berkeley CS28067 and Berkeley CS8068 were germinated and grown on solidified one-half Murashige and Skoog supplemented with the indicated concentrations of CuCl2. After (A) 10 days and (B) 20 days primary root length was measured for each treatment. Data represent the mean (N = 5; ± SE). Comparisons between accessions were done by ANOVA using Tukey (P<0.05). (TIF) [file pone.0130679.s004.tif]
