# Supplementary material for: Re-Evaluation of Reportedly Metal Tolerant Arabidopsis thaliana Accessions
Source: PLoS One. 2016 Jul 28;11(7):e0130679. doi: 10.1371/journal.pone.0130679 (PMC4965157; doi:10.1371/journal.pone.0130679)
Supplement: S1 Table — (DOCX) [file pone.0130679.s005.docx]

Table S1. Connecting letters report for zinc treatment at day 10.

| Accession | Treatment |  |  | Mean |
| --- | --- | --- | --- | --- |
| Santa Clara CS8069 | Control | A |  | 22.588864 |
| Col-0 | Control | A |  | 21.960273 |
| Santa Clara CS28722 | Control | A |  | 21.821000 |
| Limeport CS8070 | Control | A |  | 21.770042 |
| Limeport CS28464 | Control | A |  | 21.756333 |
| Berkeley CS8068 | Control | A |  | 21.402476 |
| Berkeley CS28067 | Control | A |  | 21.303600 |
| Limeport CS8070 | Zn 200µM | A |  | 20.840783 |
| Col-0 | Zn 200µM | A |  | 20.502913 |
| Santa Clara CS28722 | Zn 200µM | A |  | 20.173826 |
| Berkeley CS8068 | Zn 200µM | A |  | 20.142667 |
| Limeport CS28464 | Zn 200µM | A |  | 20.115625 |
| Santa Clara CS8069 | Zn 200µM | A |  | 20.034500 |
| Berkeley CS28067 | Zn 200µM | A |  | 19.709957 |
| Berkeley CS8068 | Zn 400µM |  | B | 6.018458 |
| Berkeley CS28067 | Zn 400µM |  | B | 5.939800 |
| Limeport CS8070 | Zn 400µM |  | B | 5.791333 |
| Limeport CS28464 | Zn 400µM |  | B | 5.667000 |
| Santa Clara CS28722 | Zn 400µM |  | B | 5.543591 |
| Col-0 | Zn 400µM |  | B | 5.520625 |
| Santa Clara CS8069 | Zn 400µM |  | B | 5.466417 |
| Col-0 | Zn 600µM |  | B | 2.464640 |
| Santa Clara CS8069 | Zn 600µM |  | B | 2.457381 |
| Berkeley CS8068 | Zn 600µM |  | B | 2.421217 |
| Limeport CS28464 | Zn 600µM |  | B | 2.372043 |
| Santa Clara CS28722 | Zn 600µM |  | B | 2.306190 |
| Berkeley CS28067 | Zn 600µM |  | B | 2.306083 |
| Limeport CS8070 | Zn 600µM |  | B | 2.022292 |

Levels not connected by same letter are significantly different (P<0.05).
