# Supplementary material for: Re-Evaluation of Reportedly Metal Tolerant Arabidopsis thaliana Accessions
Source: PLoS One. 2016 Jul 28;11(7):e0130679. doi: 10.1371/journal.pone.0130679 (PMC4965157; doi:10.1371/journal.pone.0130679)
Supplement: S2 Table — (DOCX) [file pone.0130679.s006.docx]

Table S2. Connecting letters report for zinc treatment at day 15.

| Accession | Treatment |  |  |  |  |  |  | Mean |
| --- | --- | --- | --- | --- | --- | --- | --- | --- |
| Berkeley CS8068 | Control | A | B |  |  |  |  | 49.650571 |
| Col-0 | Control | A |  |  |  |  |  | 49.476565 |
| Berkeley CS28067 | Control | A | B |  |  |  |  | 48.745739 |
| Limeport CS8070 | Control | A | B |  |  |  |  | 48.612261 |
| Limeport CS28464 | Control | A | B | C |  |  |  | 48.457524 |
| Santa Clara CS28722 | Control | A | B | C | D |  |  | 46.871000 |
| Santa Clara CS8069 | Control | A | B | C | D | E |  | 44.245667 |
| Limeport CS28464 | Zn 200µM | A | B | C | D | E |  | 32.928565 |
| Berkeley CS8068 | Zn 200µM |  | B | C | D | E |  | 32.667522 |
| Limeport CS8070 | Zn 200µM |  |  | C | D | E |  | 31.423636 |
| Berkeley CS28067 | Zn 200µM |  |  |  | D | E |  | 30.846174 |
| Santa Clara CS28722 | Zn 200µM |  |  |  | D | E |  | 30.696200 |
| Santa Clara CS8069 | Zn 200µM |  |  |  |  | E |  | 30.089043 |
| Col-0 | Zn 200µM |  |  |  |  | E |  | 30.012130 |
| Limeport CS8070 | Zn 400µM |  |  |  |  |  | F | 6.064080 |
| Col-0 | Zn 400µM |  |  |  |  |  | F | 5.836125 |
| Berkeley CS8068 | Zn 400µM |  |  |  |  |  | F | 5.638773 |
| Berkeley CS28067 | Zn 400µM |  |  |  |  |  | F | 5.593000 |
| Limeport CS28464 | Zn 400µM |  |  |  |  |  | F | 5.537409 |
| Santa Clara CS28722 | Zn 400µM |  |  |  |  |  | F | 5.470652 |
| Santa Clara CS8069 | Zn 400µM |  |  |  |  |  | F | 4.123952 |
| Col-0 | Zn 600µM |  |  |  |  |  | F | 2.854800 |
| Limeport CS28464 | Zn 600µM |  |  |  |  |  | F | 2.304045 |
| Berkeley CS28067 | Zn 600µM |  |  |  |  |  | F | 2.248409 |
| Berkeley CS8068 | Zn 600µM |  |  |  |  |  | F | 2.248409 |
| Limeport CS8070 | Zn 600µM |  |  |  |  |  | F | 2.196167 |
| Santa Clara CS28722 | Zn 600µM |  |  |  |  |  | F | 2.115053 |
| Santa Clara CS8069 | Zn 600µM |  |  |  |  |  | F | 2.010632 |

Levels not connected by same letter are significantly different (P<0.05).
