# Supplementary material for: Re-Evaluation of Reportedly Metal Tolerant Arabidopsis thaliana Accessions
Source: PLoS One. 2016 Jul 28;11(7):e0130679. doi: 10.1371/journal.pone.0130679 (PMC4965157; doi:10.1371/journal.pone.0130679)
Supplement: S3 Table — (DOCX) [file pone.0130679.s007.docx]

Table S3. Connecting letters report for zinc treatment at day 20.

| Accession | Treatment |  |  |  |  |  | Mean |
| --- | --- | --- | --- | --- | --- | --- | --- |
| Berkeley CS8068 | Control | A |  |  |  |  | 62.465043 |
| Berkeley CS28067 | Control | A |  |  |  |  | 61.584640 |
| Col-0 | Control | A | B |  |  |  | 60.000417 |
| Santa Clara CS28722 | Control | A | B |  |  |  | 57.946174 |
| Limeport CS8070 | Control | A | B |  |  |  | 57.779565 |
| Santa Clara CS8069 | Control | A | B |  |  |  | 57.507920 |
| Limeport CS28464 | Control | A | B | C |  |  | 56.639217 |
| Limeport CS28464 | Zn 200µM |  | B | C | D |  | 44.216792 |
| Berkeley CS28067 | Zn 200µM |  |  | C | D |  | 40.556440 |
| Berkeley CS8068 | Zn 200µM |  |  | C | D |  | 40.279348 |
| Santa Clara CS8069 | Zn 200µM |  |  |  | D |  | 39.172708 |
| Limeport CS8070 | Zn 200µM |  |  |  | D |  | 38.737480 |
| Santa Clara CS28722 | Zn 200µM |  |  |  | D |  | 38.053292 |
| Col-0 | Zn 200µM |  |  |  | D |  | 37.183500 |
| Berkeley CS28067 | Zn 400µM |  |  |  |  | E | 8.397320 |
| Limeport CS8070 | Zn 400µM |  |  |  |  | E | 7.645739 |
| Col-0 | Zn 400µM |  |  |  |  | E | 7.098667 |
| Berkeley CS8068 | Zn 400µM |  |  |  |  | E | 7.020120 |
| Limeport CS28464 | Zn 400µM |  |  |  |  | E | 6.551240 |
| Santa Clara CS8069 | Zn 400µM |  |  |  |  | E | 5.847160 |
| Santa Clara CS28722 | Zn 400µM |  |  |  |  | E | 5.204400 |
| Santa Clara CS28722 | Zn 600µM |  |  |  |  | E | 3.127500 |
| Limeport CS28464 | Zn 600µM |  |  |  |  | E | 2.703250 |
| Santa Clara CS8069 | Zn 600µM |  |  |  |  | E | 2.651042 |
| Col-0 | Zn 600µM |  |  |  |  | E | 2.375080 |
| Limeport CS8070 | Zn 600µM |  |  |  |  | E | 2.088833 |
| Berkeley CS28067 | Zn 600µM |  |  |  |  | E | 2.046400 |
| Berkeley CS8068 | Zn 600µM |  |  |  |  | E | 1.950958 |

Levels not connected by same letter are significantly different (P<0.05).
