# Supplementary material for: Re-Evaluation of Reportedly Metal Tolerant Arabidopsis thaliana Accessions
Source: PLoS One. 2016 Jul 28;11(7):e0130679. doi: 10.1371/journal.pone.0130679 (PMC4965157; doi:10.1371/journal.pone.0130679)
Supplement: S4 Table — (DOCX) [file pone.0130679.s008.docx]

Table S4. Connecting letters report for nickel treatment at day 10.

| Accession | Treatment |  |  |  |  |  |  |  |  |  |  |  |  | Mean |
| --- | --- | --- | --- | --- | --- | --- | --- | --- | --- | --- | --- | --- | --- | --- |
| Santa Clara CS8069 | Control | A |  |  |  |  |  |  |  |  |  |  |  | 22.588864 |
| Col-0 | Control | A | B |  |  |  |  |  |  |  |  |  |  | 21.960273 |
| Santa Clara CS28722 | Control | A | B |  |  |  |  |  |  |  |  |  |  | 21.821000 |
| Limeport CS8070 | Control | A | B |  |  |  |  |  |  |  |  |  |  | 21.770042 |
| Limeport CS28464 | Control | A | B |  |  |  |  |  |  |  |  |  |  | 21.756333 |
| Berkeley CS8068 | Control | A | B | C | D |  |  |  |  |  |  |  |  | 21.402476 |
| Berkeley CS28067 | Control | A | B | C |  |  |  |  |  |  |  |  |  | 21.303600 |
| Berkeley CS28067 | Ni 50µM | A | B | C | D | E |  |  |  |  |  |  |  | 17.857600 |
| Limeport CS28464 | Ni 50µM | A | B | C | D | E |  |  |  |  |  |  |  | 17.520348 |
| Berkeley CS8068 | Ni 50µM | A | B | C | D | E |  |  |  |  |  |  |  | 17.345238 |
| Col-0 | Ni 50µM | A | B | C | D | E |  |  |  |  |  |  |  | 17.172880 |
| Santa Clara CS8069 | Ni 50µM | A | B | C | D | E |  |  |  |  |  |  |  | 17.141333 |
| Limeport CS8070 | Ni 50µM | A | B | C | D | E |  |  |  |  |  |  |  | 17.136167 |
| Santa Clara CS28722 | Ni 50µM | A | B | C | D | E |  |  |  |  |  |  |  | 17.045714 |
| Limeport CS8070 | Ni 75µM | A | B | C | D | E | F |  |  |  |  |  |  | 15.156625 |
| Col-0 | Ni 75µM | A | B | C | D | E | F |  |  |  |  |  |  | 14.609800 |
| Berkeley CS8068 | Ni 75µM | A | B | C | D | E | F |  |  |  |  |  |  | 14.540708 |
| Santa Clara CS28722 | Ni 75µM | A | B | C | D | E | F | G |  |  |  |  |  | 14.113545 |
| Limeport CS28464 | Ni 75µM | A | B | C | D | E | F | G |  |  |  |  |  | 13.927130 |
| Santa Clara CS8069 | Ni 75µM | A | B | C | D | E | F | G | H |  |  |  |  | 13.673591 |
| Berkeley CS28067 | Ni 75µM |  | B | C | D | E | F | G | H |  |  |  |  | 13.425292 |
| Limeport CS8070 | Ni 100µM |  | B | C | D | E | F | G | H | I |  |  |  | 13.123478 |
| Limeport CS28464 | Ni 100µM |  | B | C | D | E | F | G | H | I | J |  |  | 12.848750 |
| Santa Clara CS28722 | Ni 100µM |  | B | C | D | E | F | G | H | I | J |  |  | 12.829158 |
| Berkeley CS28067 | Ni 100µM |  |  | C | D | E | F | G | H | I | J |  |  | 12.482783 |
| Santa Clara CS8069 | Ni 100µM |  |  | C | D | E | F | G | H | I | J |  |  | 12.355130 |
| Col-0 | Ni 100µM |  |  |  | D | E | F | G | H | I | J |  |  | 12.230292 |
| Berkeley CS8068 | Ni 100µM |  |  |  |  | E | F | G | H | I | J | K |  | 11.369174 |
| Limeport CS8070 | Ni 125µM |  |  |  |  |  | F | G | H | I | J | K | L | 6.795870 |
| Santa Clara CS8069 | Ni 125µM |  |  |  |  |  | F | G | H | I | J | K | L | 6.752696 |
| Limeport CS28464 | Ni 125µM |  |  |  |  |  | F | G | H | I | J | K | L | 6.558952 |
| Berkeley CS8068 | Ni 125µM |  |  |  |  |  | F | G | H | I | J | K | L | 6.296043 |
| Santa Clara CS28722 | Ni 125µM |  |  |  |  |  | F | G | H | I | J | K | L | 6.257833 |
| Col-0 | Ni 125µM |  |  |  |  |  | F | G | H | I | J | K | L | 6.212000 |
| Berkeley CS28067 | Ni 125µM |  |  |  |  |  | F | G | H | I | J | K | L | 6.118095 |
| Limeport CS8070 | Ni 150µM |  |  |  |  |  |  | G | H | I | J | K | L | 4.706050 |
| Limeport CS28464 | Ni 150µM |  |  |  |  |  |  |  |  | I | J | K | L | 4.226696 |
| Col-0 | Ni 150µM |  |  |  |  |  |  |  |  | I | J | K | L | 4.202136 |
| Santa Clara CS28722 | Ni 150µM |  |  |  |  |  |  |  |  | I | J | K | L | 4.144864 |
| Berkeley CS28067 | Ni 150µM |  |  |  |  |  |  |  |  |  | J | K | L | 4.140120 |
| Santa Clara CS8069 | Ni 150µM |  |  |  |  |  |  |  | H | I | J | K | L | 4.136800 |
| Berkeley CS8068 | Ni 150µM |  |  |  |  |  |  |  |  | I | J | K | L | 4.098136 |
| Limeport CS8070 | Ni 175µM |  |  |  |  |  |  |  |  |  |  | K | L | 3.273040 |
| Berkeley CS8068 | Ni 175µM |  |  |  |  |  |  |  |  |  |  | K | L | 2.976760 |
| Col-0 | Ni 175µM |  |  |  |  |  |  |  |  |  |  | K | L | 2.749739 |
| Berkeley CS28067 | Ni 175µM |  |  |  |  |  |  |  |  |  |  | K | L | 2.736591 |
| Santa Clara CS28722 | Ni 175µM |  |  |  |  |  |  |  |  |  |  | K | L | 2.674045 |
| Limeport CS28464 | Ni 175µM |  |  |  |  |  |  |  |  |  |  | K | L | 2.661958 |
| Santa Clara CS8069 | Ni 175µM |  |  |  |  |  |  |  |  |  |  | K | L | 2.620727 |
| Limeport CS8070 | Ni 200µM |  |  |  |  |  |  |  |  |  |  | K | L | 2.488909 |
| Santa Clara CS28722 | Ni 200µM |  |  |  |  |  |  |  |  |  |  | K | L | 2.282895 |
| Limeport CS28464 | Ni 200µM |  |  |  |  |  |  |  |  |  |  | K | L | 2.237136 |
| Col-0 | Ni 200µM |  |  |  |  |  |  |  |  |  |  |  | L | 2.196560 |
| Santa Clara CS8069 | Ni 200µM |  |  |  |  |  |  |  |  |  |  |  | L | 2.120167 |
| Berkeley CS8068 | Ni 200µM |  |  |  |  |  |  |  |  |  |  |  | L | 2.058591 |
| Berkeley CS28067 | Ni 200µM |  |  |  |  |  |  |  |  |  |  |  | L | 1.973130 |

Levels not connected by same letter are significantly different (P<0.05).
