# Supplementary material for: Re-Evaluation of Reportedly Metal Tolerant Arabidopsis thaliana Accessions
Source: PLoS One. 2016 Jul 28;11(7):e0130679. doi: 10.1371/journal.pone.0130679 (PMC4965157; doi:10.1371/journal.pone.0130679)
Supplement: S5 Table — (DOCX) [file pone.0130679.s009.docx]

Table S5. Connecting letters report for nickel treatment at day 15.

| Accession | Treatment |  |  |  |  |  |  |  |  |  |  |  | Mean |
| --- | --- | --- | --- | --- | --- | --- | --- | --- | --- | --- | --- | --- | --- |
| Berkeley CS8068 | Control | A |  |  |  |  |  |  |  |  |  |  | 49.650571 |
| Col-0 | Control | A |  |  |  |  |  |  |  |  |  |  | 49.476565 |
| Berkeley CS28067 | Control | A |  |  |  |  |  |  |  |  |  |  | 48.745739 |
| Limeport CS8070 | Control | A |  |  |  |  |  |  |  |  |  |  | 48.612261 |
| Limeport CS28464 | Control | A |  |  |  |  |  |  |  |  |  |  | 48.457524 |
| Santa Clara CS28722 | Control | A | B |  |  |  |  |  |  |  |  |  | 46.871000 |
| Santa Clara CS8069 | Control | A | B |  |  |  |  |  |  |  |  |  | 44.245667 |
| Berkeley CS8068 | Ni 50µM | A | B | C |  |  |  |  |  |  |  |  | 38.435273 |
| Limeport CS8070 | Ni 50µM | A | B | C |  |  |  |  |  |  |  |  | 38.017522 |
| Berkeley CS28067 | Ni 50µM | A | B | C |  |  |  |  |  |  |  |  | 37.905042 |
| Limeport CS8070 | Ni 75µM | A | B | C |  |  |  |  |  |  |  |  | 37.828708 |
| Santa Clara CS28722 | Ni 50µM | A | B | C |  |  |  |  |  |  |  |  | 37.373053 |
| Limeport CS28464 | Ni 50µM | A | B | C |  |  |  |  |  |  |  |  | 37.259150 |
| Santa Clara CS8069 | Ni 50µM | A | B | C |  |  |  |  |  |  |  |  | 36.654636 |
| Col-0 | Ni 50µM | A | B | C |  |  |  |  |  |  |  |  | 36.109286 |
| Limeport CS28464 | Ni 75µM | A | B | C |  |  |  |  |  |  |  |  | 33.828391 |
| Col-0 | Ni 75µM | A | B | C | D |  |  |  |  |  |  |  | 33.022875 |
| Santa Clara CS28722 | Ni 75µM | A | B | C | D | E |  |  |  |  |  |  | 32.461889 |
| Berkeley CS8068 | Ni 75µM |  | B | C | D | E |  |  |  |  |  |  | 29.376800 |
| Santa Clara CS8069 | Ni 75µM |  | B | C | D | E | F |  |  |  |  |  | 29.087609 |
| Berkeley CS28067 | Ni 75µM |  | B | C | D | E | F |  |  |  |  |  | 28.652583 |
| Limeport CS8070 | Ni 100µM |  |  | C | D | E | F | G |  |  |  |  | 23.580870 |
| Col-0 | Ni 100µM |  |  | C | D | E | F | G |  |  |  |  | 22.761783 |
| Berkeley CS28067 | Ni 100µM |  |  | C | D | E | F | G |  |  |  |  | 22.413680 |
| Santa Clara CS28722 | Ni 100µM |  |  | C | D | E | F | G | H |  |  |  | 22.108333 |
| Berkeley CS8068 | Ni 100µM |  |  | C | D | E | F | G | H | I |  |  | 21.349682 |
| Santa Clara CS8069 | Ni 100µM |  |  | C | D | E | F | G | H | I | J |  | 20.268520 |
| Limeport CS28464 | Ni 100µM |  |  | C | D | E | F | G | H | I | J | K | 20.030833 |
| Santa Clara CS8069 | Ni 125µM |  |  |  | D | E | F | G | H | I | J | K | 15.039273 |
| Limeport CS28464 | Ni 125µM |  |  |  | D | E | F | G | H | I | J | K | 14.862500 |
| Berkeley CS28067 | Ni 125µM |  |  |  |  | E | F | G | H | I | J | K | 14.189040 |
| Col-0 | Ni 125µM |  |  |  |  | E | F | G | H | I | J | K | 13.562826 |
| Santa Clara CS28722 | Ni 125µM |  |  |  |  | E | F | G | H | I | J | K | 13.141042 |
| Limeport CS8070 | Ni 125µM |  |  |  |  | E | F | G | H | I | J | K | 12.858591 |
| Berkeley CS8068 | Ni 125µM |  |  |  |  |  | F | G | H | I | J | K | 11.044000 |
| Col-0 | Ni 150µM |  |  |  |  |  |  | G | H | I | J | K | 6.702682 |
| Limeport CS8070 | Ni 150µM |  |  |  |  |  |  | G | H | I | J | K | 6.425565 |
| Santa Clara CS8069 | Ni 150µM |  |  |  |  |  |  | G | H | I | J | K | 6.160190 |
| Limeport CS28464 | Ni 150µM |  |  |  |  |  |  | G | H | I | J | K | 5.628478 |
| Berkeley CS28067 | Ni 150µM |  |  |  |  |  |  | G | H | I | J | K | 5.506920 |
| Berkeley CS8068 | Ni 150µM |  |  |  |  |  |  | G | H | I | J | K | 5.309261 |
| Santa Clara CS28722 | Ni 150µM |  |  |  |  |  |  | G | H | I | J | K | 5.192050 |
| Limeport CS8070 | Ni 175µM |  |  |  |  |  |  |  | H | I | J | K | 3.468870 |
| Berkeley CS8068 | Ni 175µM |  |  |  |  |  |  |  |  | I | J | K | 3.065750 |
| Berkeley CS28067 | Ni 175µM |  |  |  |  |  |  |  |  | I | J | K | 3.028913 |
| Santa Clara CS28722 | Ni 175µM |  |  |  |  |  |  |  | H | I | J | K | 2.898095 |
| Col-0 | Ni 175µM |  |  |  |  |  |  |  |  | I | J | K | 2.815043 |
| Limeport CS28464 | Ni 175µM |  |  |  |  |  |  |  | H | I | J | K | 2.683762 |
| Santa Clara CS8069 | Ni 175µM |  |  |  |  |  |  |  |  |  | J | K | 2.485040 |
| Limeport CS8070 | Ni 200µM |  |  |  |  |  |  |  |  |  | J | K | 2.367500 |
| Berkeley CS8068 | Ni 200µM |  |  |  |  |  |  |  |  |  | J | K | 2.339000 |
| Col-0 | Ni 200µM |  |  |  |  |  |  |  |  |  |  | K | 2.279920 |
| Santa Clara CS8069 | Ni 200µM |  |  |  |  |  |  |  |  |  | J | K | 2.207455 |
| Santa Clara CS28722 | Ni 200µM |  |  |  |  |  |  |  |  | I | J | K | 2.174905 |
| Berkeley CS28067 | Ni 200µM |  |  |  |  |  |  |  |  |  |  | K | 2.148958 |
| Limeport CS28464 | Ni 200µM |  |  |  |  |  |  |  |  |  |  | K | 1.823391 |

Levels not connected by same letter are significantly different (P<0.05).
