# Supplementary material for: Re-Evaluation of Reportedly Metal Tolerant Arabidopsis thaliana Accessions
Source: PLoS One. 2016 Jul 28;11(7):e0130679. doi: 10.1371/journal.pone.0130679 (PMC4965157; doi:10.1371/journal.pone.0130679)
Supplement: S6 Table — (DOCX) [file pone.0130679.s010.docx]

Table S6. Connecting letters report for nickel treatment at day 20.

| Accession | Treatment |  |  |  |  |  |  |  |  |  |  |  |  |  | Mean |
| --- | --- | --- | --- | --- | --- | --- | --- | --- | --- | --- | --- | --- | --- | --- | --- |
| Berkeley CS8068 | Control | A |  |  |  |  |  |  |  |  |  |  |  |  | 62.465043 |
| Berkeley CS28067 | Control | A |  |  |  |  |  |  |  |  |  |  |  |  | 61.584640 |
| Col-0 | Control | A | B |  |  |  |  |  |  |  |  |  |  |  | 60.000417 |
| Santa Clara CS28722 | Control | A | B |  |  |  |  |  |  |  |  |  |  |  | 57.946174 |
| Limeport CS8070 | Control | A | B |  |  |  |  |  |  |  |  |  |  |  | 57.779565 |
| Santa Clara CS8069 | Control | A | B |  |  |  |  |  |  |  |  |  |  |  | 57.507920 |
| Limeport CS28464 | Control | A | B |  |  |  |  |  |  |  |  |  |  |  | 56.639217 |
| Berkeley CS8068 | Ni 50µM | A | B | C |  |  |  |  |  |  |  |  |  |  | 49.204800 |
| Santa Clara CS28722 | Ni 50µM | A | B | C | D | E |  |  |  |  |  |  |  |  | 48.217524 |
| Limeport CS8070 | Ni 75µM | A | B | C | D |  |  |  |  |  |  |  |  |  | 47.750750 |
| Col-0 | Ni 50µM | A | B | C | D | E |  |  |  |  |  |  |  |  | 47.561750 |
| Berkeley CS28067 | Ni 50µM | A | B | C | D | E | F |  |  |  |  |  |  |  | 47.080640 |
| Santa Clara CS8069 | Ni 50µM | A | B | C | D | E | F |  |  |  |  |  |  |  | 47.056435 |
| Limeport CS28464 | Ni 50µM | A | B | C | D | E | F |  |  |  |  |  |  |  | 46.895435 |
| Col-0 | Ni 75µM | A | B | C | D | E | F | G |  |  |  |  |  |  | 44.974773 |
| Limeport CS8070 | Ni 50µM | A | B | C | D | E | F | G |  |  |  |  |  |  | 44.389636 |
| Limeport CS28464 | Ni 75µM | A | B | C | D | E | F | G |  |  |  |  |  |  | 43.224773 |
| Berkeley CS8068 | Ni 75µM | A | B | C | D | E | F | G |  |  |  |  |  |  | 43.125667 |
| Santa Clara CS28722 | Ni 75µM | A | B | C | D | E | F | G |  |  |  |  |  |  | 42.685261 |
| Berkeley CS28067 | Ni 75µM |  | B | C | D | E | F | G |  |  |  |  |  |  | 41.585043 |
| Santa Clara CS8069 | Ni 75µM |  | B | C | D | E | F | G |  |  |  |  |  |  | 40.456773 |
| Col-0 | Ni 100µM |  |  | C | D | E | F | G | H |  |  |  |  |  | 33.432640 |
| Limeport CS8070 | Ni 100µM |  |  | C | D | E | F | G | H | I |  |  |  |  | 31.125042 |
| Berkeley CS28067 | Ni 100µM |  |  |  | D | E | F | G | H | I |  |  |  |  | 29.843360 |
| Berkeley CS8068 | Ni 100µM |  |  | C | D | E | F | G | H | I |  |  |  |  | 29.603913 |
| Limeport CS28464 | Ni 100µM |  |  |  |  | E | F | G | H | I | J |  |  |  | 28.239600 |
| Santa Clara CS8069 | Ni 100µM |  |  |  |  |  | F | G | H | I | J | K |  |  | 27.154000 |
| Santa Clara CS28722 | Ni 100µM |  |  |  |  |  |  | G | H | I | J | K | L |  | 26.328783 |
| Berkeley CS8068 | Ni 125µM |  |  |  |  |  |  |  | H | I | J | K | L | M | 16.519500 |
| Berkeley CS28067 | Ni 125µM |  |  |  |  |  |  |  | H | I | J | K | L | M | 16.080261 |
| Limeport CS8070 | Ni 125µM |  |  |  |  |  |  |  | H | I | J | K | L | M | 15.901609 |
| Col-0 | Ni 125µM |  |  |  |  |  |  |  | H | I | J | K | L | M | 15.454000 |
| Limeport CS28464 | Ni 125µM |  |  |  |  |  |  |  | H | I | J | K | L | M | 13.542273 |
| Santa Clara CS8069 | Ni 125µM |  |  |  |  |  |  |  |  | I | J | K | L | M | 13.200792 |
| Santa Clara CS28722 | Ni 125µM |  |  |  |  |  |  |  |  | I | J | K | L | M | 12.209708 |
| Limeport CS8070 | Ni 150µM |  |  |  |  |  |  |  |  |  | J | K | L | M | 8.978750 |
| Col-0 | Ni 150µM |  |  |  |  |  |  |  |  |  |  | K | L | M | 7.408636 |
| Santa Clara CS8069 | Ni 150µM |  |  |  |  |  |  |  |  |  |  |  | L | M | 6.304409 |
| Limeport CS28464 | Ni 150µM |  |  |  |  |  |  |  |  |  |  |  | L | M | 6.036727 |
| Berkeley CS28067 | Ni 150µM |  |  |  |  |  |  |  |  |  |  |  |  | M | 5.231400 |
| Santa Clara CS28722 | Ni 150µM |  |  |  |  |  |  |  |  |  |  |  |  | M | 5.038750 |
| Berkeley CS8068 | Ni 150µM |  |  |  |  |  |  |  |  |  |  |  |  | M | 4.968714 |
| Limeport CS8070 | Ni 175µM |  |  |  |  |  |  |  |  |  |  |  |  | M | 4.002500 |
| Berkeley CS8068 | Ni 175µM |  |  |  |  |  |  |  |  |  |  |  |  | M | 3.876400 |
| Col-0 | Ni 175µM |  |  |  |  |  |  |  |  |  |  |  |  | M | 3.670391 |
| Santa Clara CS8069 | Ni 175µM |  |  |  |  |  |  |  |  |  |  |  |  | M | 3.518895 |
| Berkeley CS28067 | Ni 175µM |  |  |  |  |  |  |  |  |  |  |  |  | M | 3.421500 |
| Santa Clara CS28722 | Ni 175µM |  |  |  |  |  |  |  |  |  |  |  |  | M | 3.160143 |
| Limeport CS28464 | Ni 175µM |  |  |  |  |  |  |  |  |  |  |  |  | M | 3.119250 |
| Col-0 | Ni 200µM |  |  |  |  |  |  |  |  |  |  |  |  | M | 2.405240 |
| Berkeley CS28067 | Ni 200µM |  |  |  |  |  |  |  |  |  |  |  |  | M | 2.233280 |
| Berkeley CS8068 | Ni 200µM |  |  |  |  |  |  |  |  |  |  |  |  | M | 2.200739 |
| Limeport CS8070 | Ni 200µM |  |  |  |  |  |  |  |  |  |  |  |  | M | 2.176625 |
| Limeport CS28464 | Ni 200µM |  |  |  |  |  |  |  |  |  |  |  |  | M | 2.104053 |
| Santa Clara CS8069 | Ni 200µM |  |  |  |  |  |  |  |  |  |  |  |  | M | 2.039958 |
| Santa Clara CS28722 | Ni 200µM |  |  |  |  |  |  |  |  |  |  |  |  | M | 1.915211 |

Levels not connected by same letter are significantly different (P<0.05).
