# Supplementary material for: Re-Evaluation of Reportedly Metal Tolerant Arabidopsis thaliana Accessions
Source: PLoS One. 2016 Jul 28;11(7):e0130679. doi: 10.1371/journal.pone.0130679 (PMC4965157; doi:10.1371/journal.pone.0130679)
Supplement: S7 Table — (DOCX) [file pone.0130679.s011.docx]

Table S7. Connecting letters report for copper treatment at day 10.

| Accession | Treatment |  |  |  | Mean |
| --- | --- | --- | --- | --- | --- |
| Col-0 | Control | A |  |  | 21.960273 |
| Santa Clara CS28722 | Control | A |  |  | 21.821000 |
| Limeport CS8070 | Control | A |  |  | 21.770042 |
| Limeport CS28464 | Control | A |  |  | 21.756333 |
| Berkeley CS28068 | Control | A |  |  | 21.402476 |
| Berkeley CS80067 | Control | A |  |  | 21.303600 |
| Santa Clara CS8069 | Control | A |  |  | 21.225227 |
| Limeport CS28464 | Cu 20µM | A | B |  | 19.200391 |
| Santa Clara CS28722 | Cu 20µM | A | B |  | 18.700200 |
| Berkeley CS80067 | Cu 20µM | A | B |  | 18.689800 |
| Santa Clara CS8069 | Cu 20µM | A | B | C | 18.474476 |
| Limeport CS8070 | Cu 20µM | A | B | C | 17.793375 |
| Limeport CS8070 | Cu 40µM | A | B | C | 16.758250 |
| Col-0 | Cu 20µM | A | B | C | 16.388480 |
| Berkeley CS28068 | Cu 20µM | A | B | C | 16.181783 |
| Santa Clara CS8069 | Cu 40µM | A | B | C | 15.673478 |
| Berkeley CS80067 | Cu 40µM | A | B | C | 14.365520 |
| Col-0 | Cu 40µM | A | B | C | 14.049875 |
| Limeport CS28464 | Cu 40µM | A | B | C | 13.062500 |
| Santa Clara CS28722 | Cu 40µM | A | B | C | 12.388273 |
| Berkeley CS28068 | Cu 40µM | A | B | C | 12.162217 |
| Limeport CS8070 | Cu 60µM |  | B | C | 8.340565 |
| Berkeley CS28068 | Cu 60µM |  | B | C | 8.163958 |
| Santa Clara CS28722 | Cu 60µM |  |  | C | 7.704600 |
| Col-0 | Cu 60µM |  | B | C | 7.690636 |
| Berkeley CS80067 | Cu 60µM |  |  | C | 7.369250 |
| Santa Clara CS8069 | Cu 60µM |  |  | C | 7.257083 |
| Limeport CS28464 | Cu 60µM |  |  | C | 7.155280 |

Levels not connected by same letter are significantly different (P<0.05).
