# Supplementary material for: Re-Evaluation of Reportedly Metal Tolerant Arabidopsis thaliana Accessions
Source: PLoS One. 2016 Jul 28;11(7):e0130679. doi: 10.1371/journal.pone.0130679 (PMC4965157; doi:10.1371/journal.pone.0130679)
Supplement: S8 Table — (DOCX) [file pone.0130679.s012.docx]

Table S8. Connecting letters report for copper treatment at day 15.

| Accession | Treatment |  |  | Mean |
| --- | --- | --- | --- | --- |
| Berkeley CS28068 | Control | A |  | 49.650571 |
| Col-0 | Control | A |  | 49.476565 |
| Berkeley CS80067 | Control | A |  | 48.745739 |
| Limeport CS8070 | Control | A |  | 48.612261 |
| Limeport CS28464 | Control | A |  | 48.457524 |
| Santa Clara CS28722 | Control | A |  | 46.871000 |
| Limeport CS28464 | Cu 20µM | A |  | 45.605417 |
| Santa Clara CS8069 | Control | A |  | 44.245667 |
| Berkeley CS28068 | Cu 20µM | A |  | 43.947773 |
| Santa Clara CS28722 | Cu 20µM | A |  | 42.070250 |
| Santa Clara CS8069 | Cu 20µM | A |  | 41.753522 |
| Limeport CS8070 | Cu 20µM | A |  | 41.209619 |
| Col-0 | Cu 20µM | A |  | 40.890136 |
| Berkeley CS80067 | Cu 20µM | A |  | 39.049609 |
| Santa Clara CS28722 | Cu 40µM |  | B | 19.680130 |
| Santa Clara CS8069 | Cu 40µM |  | B | 19.173280 |
| Berkeley CS28068 | Cu 40µM |  | B | 18.777458 |
| Col-0 | Cu 40µM |  | B | 17.997652 |
| Limeport CS28464 | Cu 40µM |  | B | 17.512565 |
| Limeport CS8070 | Cu 40µM |  | B | 17.241250 |
| Berkeley CS80067 | Cu 40µM |  | B | 17.102600 |
| Limeport CS8070 | Cu 60µM |  | B | 13.930625 |
| Berkeley CS28068 | Cu 60µM |  | B | 13.570333 |
| Santa Clara CS28722 | Cu 60µM |  | B | 13.245560 |
| Limeport CS28464 | Cu 60µM |  | B | 11.260565 |
| Col-0 | Cu 60µM |  | B | 10.873840 |
| Berkeley CS80067 | Cu 60µM |  | B | 10.400375 |
| Santa Clara CS8069 | Cu 60µM |  | B | 9.405520 |

Levels not connected by same letter are significantly different (P<0.05).
