# Supplementary material for: Re-Evaluation of Reportedly Metal Tolerant Arabidopsis thaliana Accessions
Source: PLoS One. 2016 Jul 28;11(7):e0130679. doi: 10.1371/journal.pone.0130679 (PMC4965157; doi:10.1371/journal.pone.0130679)
Supplement: S9 Table — (DOCX) [file pone.0130679.s013.docx]

Table S9. Connecting letters report for copper treatment at day 20.

| Accession | Treatment |  |  |  |  |  |  | Mean |
| --- | --- | --- | --- | --- | --- | --- | --- | --- |
| Berkeley CS28068 | Control | A |  |  |  |  |  | 62.465043 |
| Berkeley CS80067 | Control | A |  |  |  |  |  | 61.584640 |
| Col-0 | Control | A |  |  |  |  |  | 60.000417 |
| Santa Clara CS28722 | Control | A |  |  |  |  |  | 57.946174 |
| Limeport CS8070 | Control | A |  |  |  |  |  | 57.779565 |
| Santa Clara CS8069 | Control | A |  |  |  |  |  | 57.507920 |
| Limeport CS28464 | Control | A |  |  |  |  |  | 56.639217 |
| Col-0 | Cu 20µM | A |  |  |  |  |  | 50.166417 |
| Limeport CS28464 | Cu 20µM | A |  |  |  |  |  | 49.524417 |
| Berkeley CS28068 | Cu 20µM | A | B |  |  |  |  | 47.671208 |
| Berkeley CS80067 | Cu 20µM | A | B |  |  |  |  | 47.335125 |
| Limeport CS8070 | Cu 20µM | A | B | C |  |  |  | 47.023167 |
| Santa Clara CS8069 | Cu 20µM | A | B | C | D |  |  | 45.852565 |
| Santa Clara CS28722 | Cu 20µM | A | B | C | D | E |  | 43.114500 |
| Santa Clara CS28722 | Cu 40µM |  | B | C | D | E | F | 27.175087 |
| Col-0 | Cu 40µM |  |  | C | D | E | F | 25.685182 |
| Santa Clara CS8069 | Cu 40µM |  |  |  | D | E | F | 25.472130 |
| Limeport CS28464 | Cu 40µM |  |  |  | D | E | F | 25.254167 |
| Berkeley CS80067 | Cu 40µM |  |  |  |  | E | F | 24.674720 |
| Limeport CS8070 | Cu 40µM |  |  |  |  | E | F | 24.628160 |
| Berkeley CS28068 | Cu 40µM |  |  |  |  | E | F | 23.411478 |
| Santa Clara CS28722 | Cu 60µM |  |  |  |  |  | F | 19.946880 |
| Limeport CS8070 | Cu 60µM |  |  |  |  |  | F | 19.311200 |
| Limeport CS28464 | Cu 60µM |  |  |  |  |  | F | 18.373000 |
| Col-0 | Cu 60µM |  |  |  |  |  | F | 16.432042 |
| Berkeley CS80067 | Cu 60µM |  |  |  |  |  | F | 15.592261 |
| Santa Clara CS8069 | Cu 60µM |  |  |  |  |  | F | 15.471640 |
| Berkeley CS28068 | Cu 60µM |  |  |  |  |  | F | 13.541040 |

Levels not connected by same letter are significantly different (P<0.05).
