# Supplementary material for: Re-Evaluation of Reportedly Metal Tolerant Arabidopsis thaliana Accessions
Source: PLoS One. 2016 Jul 28;11(7):e0130679. doi: 10.1371/journal.pone.0130679 (PMC4965157; doi:10.1371/journal.pone.0130679)
Supplement: S10 Table — (DOCX) [file pone.0130679.s014.docx]

Table S10. All non-synonymous changes unique to the Limeport accessions

| Chr | Position | Ref | Lim | Gene | Impact | Codon | AA | Gene description |
| --- | --- | --- | --- | --- | --- | --- | --- | --- |
| 1 | 9123840 | C | T | AT1G26370.1 | M | Gtt/Att | V342I | RNA helicase family protein |

Chr: chromosome; Ref: nucleotide on reference sequence; Lim: nucleotide on Limeport sequence; M: moderate impact; H: high impact; *: stop codon. The codon column shows the codon for the reference and Limeport sequence at specific position separately by a slash. AA column represents the amino acid encoded on the reference codon, the protein position of the amino acid and the amino acid encoded on the Limeport codon.
