# Supplementary material for: Re-Evaluation of Reportedly Metal Tolerant Arabidopsis thaliana Accessions
Source: PLoS One. 2016 Jul 28;11(7):e0130679. doi: 10.1371/journal.pone.0130679 (PMC4965157; doi:10.1371/journal.pone.0130679)
Supplement: S11 Table — (DOCX) [file pone.0130679.s015.docx]

Table S11. All non-synonymous changes unique to the Santa Clara accessions

| Chr | Position | Ref | SC | Gene | Impact | Codon | AA | Gene description |
| --- | --- | --- | --- | --- | --- | --- | --- | --- |
| 2 | 5342127 | G | A | AT2G12990.1 | H | Cag/Tag | Q190* | Transposable element gene |
| 4 | 2231989 | C | G | AT4G04490.1 | M | Gag/Cag | E649Q | Cysteine-rich RLK (RECEPTOR-like protein kinase) 36 (CRK36) |
| 4 | 2916422 | G | A | AT4G05600.1 | M | Gcg/Acg | A12T | Transposable element gene |
| 5 | 26716017 | A | C | AT5G66900.1 | M | cTt/cGt | L487R | Disease resistance protein (CC-NBS-LRR class) family |

Chr: chromosome; Ref: nucleotide on reference sequence; SC: nucleotide on Santa Clara sequence; M: moderate impact; H: high impact; *: stop codon. The codon column shows the codon for the reference and Santa Clara sequence at specific position separately by a slash. AA column represents the amino acid encoded on the reference codon, the protein position of the amino acid and the amino acid encoded on the Santa Clara codon.
