# Supplementary material for: Re-Evaluation of Reportedly Metal Tolerant Arabidopsis thaliana Accessions
Source: PLoS One. 2016 Jul 28;11(7):e0130679. doi: 10.1371/journal.pone.0130679 (PMC4965157; doi:10.1371/journal.pone.0130679)
Supplement: S12 Table — (DOCX) [file pone.0130679.s016.docx]

Table S12. All non-synonymous changes unique to the Berkeley accessions.

| Chr | Position | Ref | Berk | Gene | Impact | Codon | AA | Gene description |
| --- | --- | --- | --- | --- | --- | --- | --- | --- |
| 2 | 782633 | T | C | AT2G02780.1 | M | Atc/Gtc | I588V | Leucine-rich repeat protein kinase family protein |
| 4 | 5690224 | C | T | AT4G08880.1 | M | Cct/Tct | P1240S | Transposable element gene |

Chr: chromosome; Ref: nucleotide on reference sequence; Berk: nucleotide on Berkeley sequence; M: moderate impact; H: high impact; *: stop codon. The codon column shows the codon for the reference and Berkeley sequence at specific position separately by a slash. AA column represents the amino acid encoded on the reference codon, the protein position of the amino acid and the amino acid encoded on the Berkeley codon.
